# Association of Coronary Artery Disease and Metabolic Syndrome: Usefulness of Serum Metabolomics Approach

**DOI:** 10.3389/fendo.2021.692893

**Published:** 2021-09-24

**Authors:** Ziwei Jing, Liwei Liu, Yingying Shi, Qiuzheng Du, Dingding Zhang, Lihua Zuo, Shuzhang Du, Zhi Sun, Xiaojian Zhang

**Affiliations:** ^1^ Department of Pharmacy, The First Affiliated Hospital of Zhengzhou University, Zhengzhou, China; ^2^ Department of Vasculocardiology, The First Affiliated Hospital of Zhengzhou University, Zhengzhou, China

**Keywords:** coronary artery disease, metabolic syndrome, UHPLC-Q-Orbitrap HRMS, serum metabolomics, clinical risk factors

## Abstract

**Introduction:**

Individuals with metabolic syndrome (MetS) are at increasing risk of coronary artery disease (CAD). We investigated the common metabolic perturbations of CAD and MetS *via* serum metabolomics to provide insight into potential associations.

**Methods:**

Non-targeted serum metabolomics analyses were performed using ultra high-performance liquid chromatography coupled with Q Exactive hybrid quadrupole-orbitrap high-resolution accurate mass spectrometry (UHPLC-Q-Orbitrap HRMS) in samples from 492 participants (272 CAD *vs.* 121 healthy controls (HCs) as cohort 1, 55 MetS *vs.* 44 HCs as cohort 2). Cross-sectional data were obtained when the participants were recruited from the First Affiliated Hospital of Zhengzhou University. Multivariate statistics and Student’s t test were applied to obtain the significant metabolites [with variable importance in the projection (VIP) values >1.0 and p values <0.05] for CAD and MetS. Logistic regression was performed to investigate the association of identified metabolites with clinical cardiac risk factors, and the association of significant metabolic perturbations between CAD and MetS was visualized by Cytoscape software 3.6.1. Finally, the receiver operating characteristic (ROC) analysis was evaluated for the risk prediction values of common changed metabolites.

**Results:**

Thirty metabolites were identified for CAD, mainly including amino acids, lipid, fatty acids, pseudouridine, niacinamide; 26 metabolites were identified for MetS, mainly including amino acids, lipid, fatty acids, steroid hormone, and paraxanthine. The logistic regression results showed that all of the 30 metabolites for CAD, and 15 metabolites for MetS remained significant after adjustments of clinical risk factors. In the common metabolic signature association analysis between CAD and MetS, 11 serum metabolites were significant and common to CAD and MetS outcomes. Out of this, nine followed similar trends while two had differing directionalities. The nine common metabolites exhibiting same change trend improved risk prediction for CAD (86.4%) and MetS (90.9%) using the ROC analysis.

**Conclusion:**

Serum metabolomics analysis might provide a new insight into the potential mechanisms underlying the common metabolic perturbations of CAD and MetS.

## Introduction

Nowadays, the significant increase of coronary artery disease (CAD) populations has become a serious challenge all over the world (1). According to the Global Burden of Disease Study 2016 (2), the incidence and years lived with disability (YLDs) of cardiovascular diseases were 54.1 million and 33.5 million that year, respectively. Interestingly, the risk factor-adjusted proportional-hazards regression of CAD mortality was doubled for the subjects with metabolic syndrome (MetS) in a 13-year follow-up report (3). Using the Framingham database, the age-adjusted relative risks for CAD with the MetS were 2.54 and 1.54 in men and women, respectively (4). If the association of CAD and MetS can be explicated, its occurrence and progression might be predicted. However, the association of CAD and MetS remains ambiguous.

Multiple and complex molecular events characterize CAD, which mainly refers to the myocardial dysfunction and/or organic lesions caused by coronary artery stenosis and insufficient blood supply (5, 6). Meanwhile, the underlying mechanism of MetS is also complicated depending on its own diverse features. According to the American Heart Association/National Heart, Lung and Blood Institute (AHA/NHLBI) criteria, metabolic syndrome is a clustering of cardiovascular disease risk factors, which include abdominal adiposity, insulin resistance, inflammations, genetic factor, abnormal neuroendocrine, unhealthy lifestyle, intrauterine malnutrition, and so on. Among these factors, abdominal adiposity and insulin resistance play a key role in the incidence of MetS (7–9). To be clear, there is a general consensus that cardiac risk factors should be aggressively managed in individuals with MetS (10).

Metabolomics is powerful omics technology that has been widely used for the unbiased identification of metabolic alterations of diseases. Metabolites could clarify the common metabolic perturbations of CAD and MetS. Fan et al. (11) reported 12 panels of metabolomics-based biomarkers in various clinical subgroups of CAD. Eighty-nine different metabolites were identified, and the altered metabolic pathways included downregulated phospholipid catabolism, tricarboxylic acid cycle, biosynthesis of primary bile acid, and upregulated amino acid metabolism, short-chain acylcarnitines. Another study exhibited the similar alterations in the gut microbiota and serum metabolites in different CAD subgroups (12). Besides, a 90 plasma-based metabolomics studies of MetS showed tyrosine, alanine, and propionylcarnitine increased and asparagine, tryptophan/large neutral amino acid ratio decreased (13). Nevertheless, serum metabolomics analysis to explore the common metabolic perturbations of CAD and MetS is still lacking.

Therefore, this work employs a serum metabolomic approach to describe metabolic alterations of CAD and MetS by ultra-high performance liquid chromatography coupled with Q Exactive hybrid quadrupole-orbitrap high-resolution mass spectrometry (UHPLC-Q-Orbitrap HRMS). The study’s specific objectives were as follows: (i) to identify metabolic signatures associated with CAD and MetS; (ii) to reveal the association of metabolic perturbations between CAD and MetS; (iii) to explore the risk predictive performance of common changed metabolites of CAD and MetS. To our knowledge, this study is the first time to explore the common metabolic perturbations of CAD and MetS by metabolomics, which could provide an insight into potential associations of the two diseases.

## Materials and Methods

### Reagents and Chemicals

The HPLC-grade methanol and acetonitrile were obtained from Fisher Scientific (Fair Lawn, NJ, USA). HPLC-grade formic acid was acquired from Aladdin Biochemical Technology Co., Ltd. (Shanghai, China). HPLC-grade H_2_O was prepared by the Millipore system (Shanghai, China). The internal standards and all the metabolite standards used in the method were purchased from J&K Scientific Ltd. (Beijing, China) and Sigma-Aldrich (St. Louis, MO, USA). Each standard substance was dissolved in methanol and prepared into a mixed standards solution (1.0 μg/ml for each compound).

### Study Design and Participants

A total of 492 participants, including 272 CAD, 55 MetS patients, and 165 HCs, were recruited from the First Affiliated Hospital of Zhengzhou University. CAD groups were diagnosed according to “Nomenclature and diagnostic criteria of ischemic heart disease” by International Society and Federation of Cardiology (ISFC) combined with World Health Organization (WHO) (14), which included chest pain symptoms, cardiovascular risk factors, pathological Q-wave on ECG, or elevated myocardial enzymes. Coronary angiography was simultaneously applied to confirm the diagnosis. MetS was diagnosed by the Chinese Diabetes Society (CDS) criteria (15), which satisfied three or more of the following index: overweight or obese [Body mass index (BMI) ≥25.0]; hyperglycemia [fasting plasma glucose (FPG) ≥6.1 mmol/L and/or plasma glucose (PG) ≥7.8 mmol/L after 2 h]; hypertension [systolic blood pressure (SBP)/diastolic blood pressure (DBP) ≥140/90 Hg]; dyslipidemia [triacylglycerol (TG) ≥1.7 mmol/L and/or high-density lipoprotein cholesterol (HDL-C) <0.9 mmol/L for men or <1.0 mmol/L for women]. The HCs were matched for age, sex, and BMI, and several simple overweight or obese people who were “healthy obese” were also included. Details of inclusion and exclusion criteria are in the **Supplementary Table 1**.

### Biochemical and Other Measures at Baseline

Clinical characteristics of the 492 participants were recorded, such as sex, age, height, weight. BMI was calculated as kg/m^2^. An automated validated device was used to measure sitting brachial blood pressure after a 10-min rest. HbA_1c_ levels were measured by a Bio-Rad Variant II hemoglobin testing system, and FPG data were recorded. The alanine aminotransferase (ALT) and aspartate transaminase (AST) levels were measured by a sandwich enzyme-linked immunosorbent assay (ELISA) system. Plasma total cholesterol (TC), triglycerides (TG), low-density lipoprotein cholesterol (LDL-C), high-density lipoprotein cholesterol (HDL-C), and serum creatinine were measured using standardized methods from venous samples. Glomerular filtration rate (eGFR) was calculated from serum creatinine using the Chronic Kidney Disease Epidemiology Collaboration (CKD-EPI) equation.

### Sample Collection and Preparation

The blood samples were collected in Na2 EDTA tubes. Each sample was centrifuged at 3,000 rpm for 10 min at 4°C to obtain supernatant. A 100 μl serum of each sample was then precipitated with 300 μl methanol containing 500 ng/ml ketoprofen and 50 ng/ml 2-chloro-L-phenylalanine as internal standard. The mixture was vortexed for 1 min followed by centrifugation at 13,000 rpm for 10 min at 4°C. Then, a 200 μl aliquot of supernatant was transferred into vials for analysis. For the assessment in parallel of reproducibility and stability along the run, quality control (QC) samples were obtained by mixing all serum samples, respectively. One QC sample was injected after every 10 samples throughout the run.

### UHPLC-Q-Orbitrap HRMS for Serum Metabolomics

A 5 μl aliquot of the prepared sample was injected into ACQUITY UPLC BEH C18 column (100 mm × 2.1 mm, 1.7 mm, Waters, USA) maintained at 40°C using a Thermo Scientific Dionex Ultimate 3000 UHPLC system for chromatographic separation. The mobile phase consisted of water containing 0.1% (v/v) formic acid (A) and acetonitrile (B). The gradient elution was set as follows at a flow rate of 0.35 ml/min: 0–1 min, 5% B; 1–9 min, 5–100% B; 9–12 min, 100% B; 12–12.1 min, 100–5% B; 12.1–15 min, 5% B.

The mass spectrometry was performed on Q-Exactive orbitrap system (Thermo Fisher Scientific, San Jose, USA) equipped with a heated electrospray ionization source operated in positive ion modes. The MS parameters were optimized and set as follows: collision energy at 20, 40, and 60 eV, ion source temperature at 350°C, capillary temperature at 320°Ct, spray voltage at 3.50 kV, sheath gas flow rate at 40 arb, auxiliary gas flow rate at 10 arb. Metabolomic profiles were acquired with a mass range of 80–1,200 m/z. The full scan spectra and MS/MS data were collected with the resolution of 70,000 and 17,500 FWHM, respectively. The samples were injected in random order, and all the mass data were acquired and processed using Thermo Xcalibur 3.0 software.

### Data Processing and Statistical Analysis

The mass spectrometry raw data were conducted by Compound Discoverer 2.1 software (Thermo Fisher Scientific, San Jose, USA). The spectra were selected from input LC-MS data files, and retention time alignment was accomplished based on mass tolerance and time shift criteria. Preliminary identification of metabolites was realized by searching databases including ChemSpider, Mass Lists, mzCloud, and mzVault. Multiple nodes such as “Align Retention Times”, “Detect Unknown Compounds”, “Group Unknown Compounds”, “Predict Compositions”, “Fill Gaps”, and “Normalize Areas” were combined to form an untargeted metabolomics workflow for raw data processing (**Supplementary Figure 1**).

The data matrix obtained from Compound Discoverer was imported into SIMICA 14.1 software (Umetrics AB, Umea, Sweden) for multivariate statistical analysis, including unsupervised principal component analysis (PCA), supervised partial least-squares discriminant analysis (PLS-DA), and orthogonal partial least-squares discriminant analysis (OPLS-DA). The PCA analysis was applied to assess the reproducibility and stability of QC samples, and the PLS-DA model was established to describe general separation of samples from different groups. The variables responsible for the discrimination between two groups were identified by OPLS-DA, and permutation test was performed 200 times to assess the risk of overfitting for the OPLS-DA model. In addition to the multivariate statistical method, the p value was calculated by Student’s t-test and adjusted by the Benjamini-Hochberg method (16). The metabolites with variable importance in the projection (VIP) values >1.0 and p values <0.05 were screened as significant altered metabolites for CAD and MetS, respectively, the structures of which were further confirmed based on available reference standards.

Logistic regression was performed to investigate whether the significant metabolites for CAD and MetS were independent to clinical cardiac risk factors. Firstly, the spectra area of the metabolites and the clinical characteristics were subjected to MetaboAnalyst 4.0 for log transformation and pareto scaling to acquire the normalized data under the same dimension. Then the odds ratios (ORs) and 95% confidence interval were obtained adjusting for three models (Model 1: adjustment for BMI; model 2: adjustment for TC, TG, LDL-C, and HDL-C; model 3: further adjustment for BMI, TC, TG, HDL-C, LDL-C, SDP, DBP, FGP, HbA1c); the workflows were shown in **Supplementary Figure 2**. Sample-size calculation was done to assess the sample size needed for replicating the metabolic signatures associations of CAD with clinical risk factors (http://www.powerandsamplesize.com/).

For association analysis of the metabolic perturbations of CAD and MetS, the global comparison of 30 significant metabolites for CAD and 26 significant metabolites for MetS were conducted. The different metabolites were divided according to the disturbed pathways. The association of significant metabolic perturbations between CAD and MetS was visualized by Cytoscape software 3.6.1. As well, the fold change values described above were regarded as the trends of metabolites levels variation.

For further confirmation of the association of CAD and MetS, the binary logistic regression and ROC analysis were performed to explore the risk predictive performance of the common changed metabolites (17, 18). The covariates of significant metabolites with same change trend were used as biomarkers panel. Based on the protocol, logistic regression was proceeded after log transformation and pareto scaling of the spectra area of 14 biomarkers to obtain the p value, then the AUC under the classical univariate ROC curve was computed using biomarker analysis of MetaboAnalyst 4.0.

## Results

**Figure 1:**
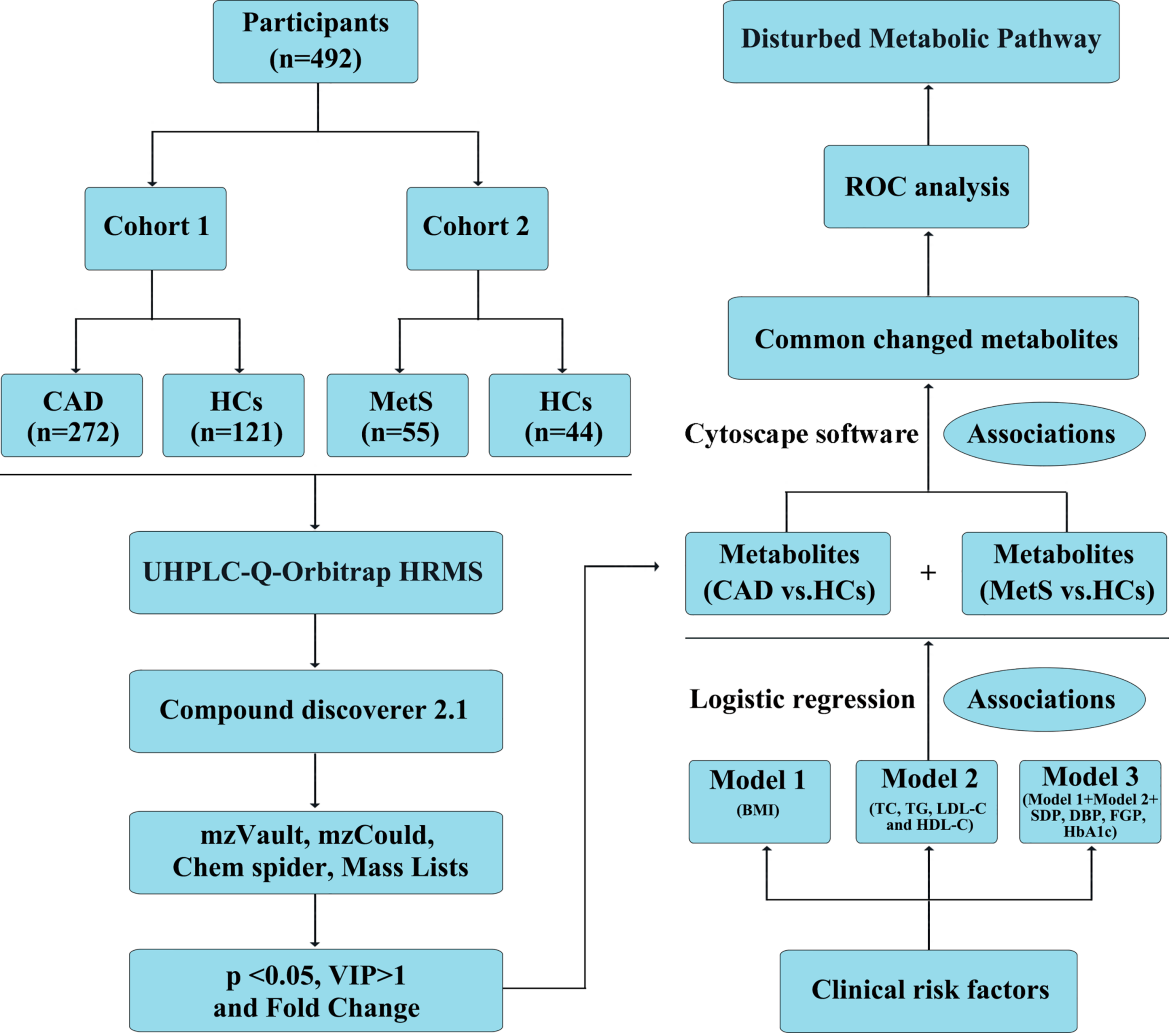
Definition and design of the study. CAD, coronary artery disease (n = 272); MetS, metabolic syndrome (n = 55); HCs, healthy controls (n = 165). Model 1: adjustment for BMI; model 2: adjustment for TC, TG, LDL-C, and HDL-C; model 3: further adjustment for BMI, TC, TG, HDL-C, LDL-C, SDP, DBP, FGP, HbA1c; Association 1: Logistic regression to investigate the association of identified metabolites with clinical cardiac risk factors; Association 2: Cytoscape software 3.6.1 to visualize the association of significant metabolites identified from CAD *vs.* HCs and MetS *vs.* HCs.

A total of 492 participants were allocated to two cohorts. As shown in **Figure 1**, 272 CAD patients and 121 HCs formed cohort 1 and 55 MetS patients and 44 HCs formed cohort 2. **Supplementary Table 2** showed the clinical characteristics and biochemical index of all the participants in this study. For CAD *vs.* HCs in cohort 1, age, sex, BMI, DBP, ALT, AST, TC, eGFR, and CR were comparable among the two populations (*p* > 0.05). However, compared with HCs, individuals with CAD had higher SBP, HbA_1c_, FPG, TG, and LDL-C, but lower HDL-C levels. Similarly, for MetS *vs.* HCs in cohort 2, MetS patients had higher SBP, HbA_1c_, FPG, TC, TG, and LDL-C, but lower levels of HDL-C. No obvious significant difference of age, sex, BMI, DBP, ALT, AST, eGFR, and CR was found between MetS and HCs.

### Metabolic Signatures Associated With CAD

**Figure 2:**
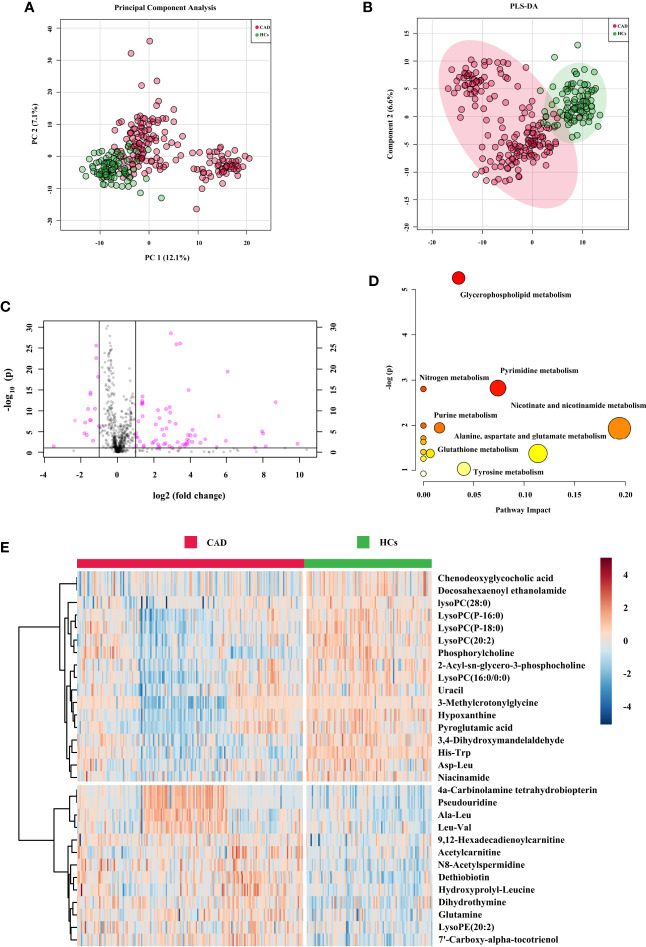
Metabolic signatures associated with CAD in the cohort 1. **(A)** The principal component analysis (PCA) compared for CAD (n = 272) to HCs (n = 121). **(B)** The partial least squares discrimination analysis (PLS-DA) compared for CAD to HCs. **(C)** The volcano plot represents the variation of metabolites amount between CAD and HCs according to the –log (p value). **(D)** Main disturbed pathways identified for MetS. Color of the circle means the metabolites are in the data with different levels of significance, with yellow being the least and red being the most significant and the range of significance being 0.1 to 1×10^−5^. Size of the circle means pathway impact values from the pathway topology analysis; the range of the circle size impact values is 0.00 to 0.20. **(E)** The heatmap represents the most significant metabolites of CAD after one-way ANOVA and hierarchical clustering of the samples.

The obtained typical total ion chromatograms (TIC) from the three representative populations are shown in **Supplementary Figure 3**. For the comparison of CAD *vs.* HCs in the cohort 1, a total of 1,673 features from positive mode were detected, and 933 ions showed significantly changed with p < 0.05. PCA, PLS-DA, and OPLS-DA score plots were performed to identify the differences of the metabolic profiles between CAD and HCs, which showed remarkable separations with cumulative R^2^Y at 0.873 and Q^2^ at 0.749 (**Figures 2A, B** and **Supplementary Figure 4**). The volcano plot represents the variation of metabolites amount for CAD *vs.* HCs according to the -log2 (fold change) (**Figure 2C**). In total, 30 identified metabolites showed significant by filtered with VIP > 1 and adjusted p values < 0.05. The information of the significant metabolites is presented in **Table 1**. And the altered pathways are shown in **Figure 2D**. The heatmap for the different metabolites in CAD and HCs is exhibited in **Figure 2E**. Combining with abundance comparison (**Supplementary Figure 5**), we found that the CAD patients had significantly different metabolite profiles compared with HCs. Of the 30 metabolites, most amino acids, lipid, primary bile acid, short-chain acylcarnitines, and purine were similar with the previous study (13, 14). The novel findings—such as pseudouridine and dihydrothymine belong to pyrimidine, niacinamide belongs to nicotinate and nicotinamide metabolism, 4a-Carbinolamine tetrahydrobiopterin belongs to folate—have not been reported previously.

**Table 1:** Significant metabolites in CAD and MetS.

Metabolites	RT (min)	Molecular Weight	Fold Change	VIP	p Value	FDR	Trend
**CAD *vs.* HCs**							
Ala-Leu	1.889	202.132	2.599	1.424	5.14E-09	1.19E-08	↑↑
4a-Carbinolamine tetrahydrobiopterin	6.419	239.231	2.597	1.519	1.27E-10	3.81E-10	↑↑
Pseudouridine	6.757	244.068	2.576	1.492	4.64E-10	1.16E-09	↑↑
7’-Carboxy-alpha-tocotrienol	8.641	346.461	1.681	1.497	2.17E-11	7.23E-11	↑↑
Leu-Val	3.434	230.163	1.565	1.004	0.000606	0.000606	↑↑
9,12-Hexadecadienoylcarnitine	7.340	395.303	1.498	1.001	3.29E-06	5.19E-06	↑
N8-Acetylspermidine*	0.921	187.168	1.438	1.558	1.69E-10	4.61E-10	↑
Dihydrothymine	0.951	128.058	1.377	1.050	1.65E-05	0.000020625	↑
Hydroxyprolyl-Leucine	1.384	244.288	1.369	1.032	1.16E-05	1.58E-05	↑
Dethiobiotin	1.385	214.131	1.363	1.538	1.02E-11	3.82E-11	↑
LysoPE (20:2)	9.113	505.316	1.338	1.071	5.21E-05	6.01E-05	↑
Acetylcarnitine*	1.380	203.115	1.217	1.056	5.13E-05	0.00006156	↑
Glutamine*	1.141	146.069	1.139	1.274	2.47E-07	4.36E-07	↑
Uracil*	1.391	112.027	0.903	1.014	0.000232	0.00024	↓
Pyroglutamic acid*	1.386	129.042	0.836	1.381	3.81E-08	7.62E-08	↓
LysoPC (P-16:0)	8.948	479.337	0.797	1.658	3.53E-12	2.12E-11	↓
asp-leu	2.414	246.121	0.796	1.234	6.39E-07	0.000001065	↓
LysoPC (16:0/0:0)	8.529	495.332	0.794	1.710	5.33E-13	3.99E-12	↓
lysoPC (28:0)	0.943	663.519	0.780	1.073	6.61E-06	0.000009915	↓
LysoPC (20:2)	8.998	547.363	0.779	1.548	9.8E-12	4.20E-11	↓
Phosphorylcholine	8.254	183.066	0.778	1.836	2.95E-13	2.95E-12	↓
2-Acyl-sn-glycero-3-phosphocholine	8.901	284.223	0.740	1.281	4.82E-08	9.04E-08	↓
LysoPC (P-18:0)	9.837	507.368	0.728	1.667	7.45E-12	3.72E-11	↓
Niacinamide	1.401	122.048	0.695	1.286	8.8E-06	1.26E-05	↓
3-Methylcrotonylglycine	1.381	157.074	0.687	1.132	1.43E-05	1.87E-05	↓
Hypoxanthine*	1.358	136.038	0.620	2.333	2.32E-21	3.48E-20	↓↓
Chenodeoxyglycocholic acid	6.530	449.313	0.591	1.153	5.42E-05	5.81E-05	↓↓
Docosahexaenoyl Ethanolamide	7.304	371.556	0.568	1.147	5.28E-05	5.87E-05	↓↓
His-Trp	3.199	341.148	0.447	2.267	1.58E-24	4.74E-23	↓↓
3,4-Dihydroxymandelaldehyde	4.240	168.147	0.360	1.463	2.45E-08	5.25E-08	↓↓
**MetS *vs.* HCs**							
Niacinamide	1.401	122.048	2.267	1.631	5.93E-06	0.0001779	↑↑
Acetylcarnitine*	1.380	203.115	1.248	1.105	0.003739	0.00467375	↑
Lysine*	0.777	146.105	0.884	1.137	0.002171	0.003101429	↓
LysoPC (16:0/0:0)	8.529	495.332	0.865	1.357	0.000534	0.00100125	↓
Uracil*	1.391	112.027	0.855	1.087	0.006973	0.008045769	↓
Sphingosine 1-phosphate	7.734	379.248	0.848	1.273	0.002011	0.0030165	↓
2-Acetyl-1-alkyl-sn-glycero-3-phosphocholine	9.375	523.363	0.845	1.249	0.002491	0.003396818	↓
Phosphorylcholine	8.254	183.066	0.844	1.464	0.000166	0.000452727	↓
Dihydrothymine	0.951	128.058	0.798	1.269	0.000836	0.00132	↓
Valine*	1.352	117.079	0.797	1.361	0.000333	0.000768462	↓
Tyrosine*	1.437	164.047	0.796	1.477	3.39E-05	0.000145286	↓
LysoPC (P-16:0)	8.948	479.337	0.792	1.589	5.61E-05	0.000187	↓
Ceramide 1-phosphate	8.547	505.352	0.771	1.600	6.37E-05	0.0001911	↓
Ornithine*	0.776	132.090	0.770	1.381	0.000253	0.0006325	↓
Citrulline*	0.917	175.095	0.764	1.334	0.000464	0.000928	↓
Proline*	0.941	115.063	0.763	1.102	0.003577	0.004665652	↓
PC (18:1 (9Z)e/2:0)	9.709	549.378	0.759	1.425	0.000563	0.000993529	↓
Pyroglutamic acid*	1.386	129.042	0.746	1.722	1.81E-06	0.00000905	↓
2-Acyl-sn-glycero-3-phosphocholine	8.901	284.223	0.724	1.918	7.16E-07	0.00000537	↓
Kynurenine	2.592	191.058	0.722	1.564	4.69E-05	0.000175875	↓
Cortisol	5.910	362.208	0.646	1.810	7.67E-07	0.000004602	↓↓
Tryptophan*	3.669	408.179	0.601	1.900	1.99E-07	0.00000199	↓↓
Tetrahydrocorticosterone	6.853	367.271	0.599	1.389	0.000667	0.001111667	↓↓
Hypoxanthine*	1.358	136.038	0.585	2.223	8.67E-11	1.30E-09	↓↓
Chenodeoxyglycocholic acid	6.530	449.313	0.413	1.197	0.003739	0.0044868	↓↓
Paraxanthine*	3.636	180.064	0.249	1.378	0.000365	0.000782143	↓↓

*Metabolites matched with commercial available reference standards; RT, retention time; Fold change, CAD/HCs in cohort 1 or MetS/HCs in cohort 2; VIP, variable importance in the project obtained from CAD vs. HCs or MetS vs. HCs. FDR, the value was obtained from the false discovery rate (FDR) correction using Benjamini-Hochberg method.

↑↑: fold change >1.5; ↑: 1 < fold change <1.5; ↓: 0.67 < fold change <1; ↓↓: fold < 0.67.

### Metabolic Signatures Associated With MetS

**Figure 3:**
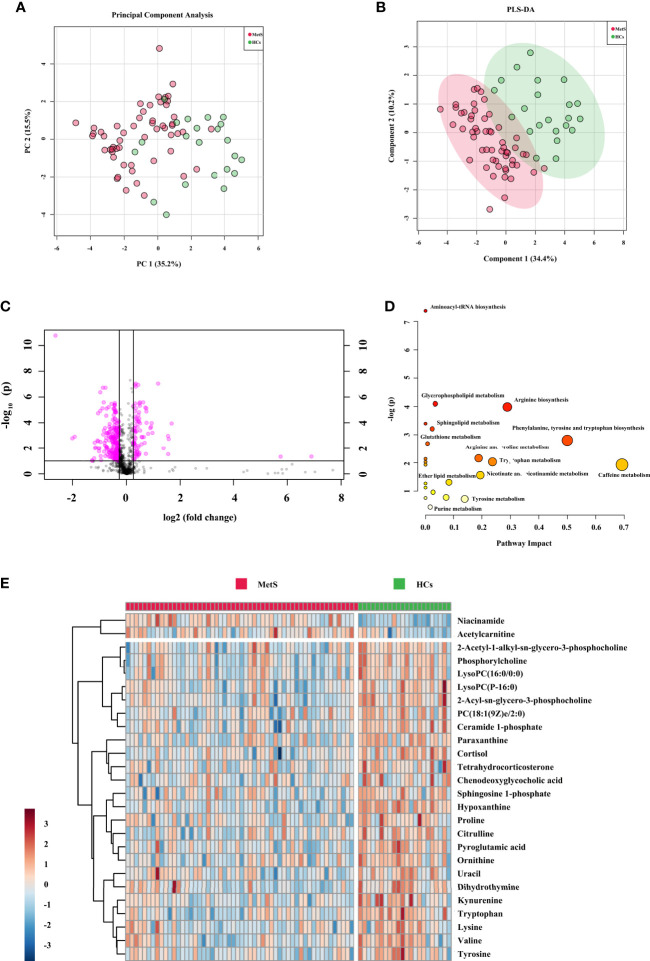
Metabolic signatures associated with MetS in the cohort 2. **(A)** The principal component analysis (PCA) compared for MetS (n = 55) to HCs (n = 44). **(B)** The partial least squares discrimination analysis (PLS-DA) compared for MetS to HCs. **(C)** The volcano plot represents the variation of metabolites amount between MetS and HCs according to the –log (p value). **(D)** Main disturbed pathways identified for MetS. The color of the circle means the metabolites are in the data with different levels of significance, with yellow being the least and red being the most significant and the range of significance being 0.1 to 1×10^−7^. The size of the circle means pathway impact values from the pathway topology analysis; the range of the circle size impact values is 0.00 to 0.70. **(E)** The heatmap represents the most significant metabolites of MetS after one-way ANOVA and hierarchical clustering of the samples.

For the comparison of MetS *vs.* HCs in the cohort 2, a total of 920 features from positive mode were detected, and 475 ions showed significantly changed (p < 0.05). After further filtering by VIP > 1 from PCA, PLS-DA, and OPLS-DA score plots (**Figures 3A, B** and **Supplementary Figure 6**), 26 identified metabolites showed significant. The OPLS-DA showed remarkable separations with cumulative R^2^Y at 0.978 and Q^2^ at 0.701. **Figure 3C** exhibited volcano plot of the variation of metabolites amount for MetS *vs.* HCs. And the pathways were showed in **Figure 3D**. The heatmap for the different metabolites in MetS and HCs was exhibited in **Figure 3E**. The information of the 26 significant metabolites is presented in **Table 1**. Combining with abundance comparison (**Supplementary Figure 7**), we found that most of the metabolism descended for amine acids, lipids, purine, pyrimidine, primary bile acid, and steroid hormone, except for elevated fatty acids and niacinamide. Compared to a previous study (13, 19), new metabolites for MetS, including uracil, hypoxanthine and paraxanthine, dihydrothymine, niacinamide, cortisol, and tetrahydrocorticosterone, have not been reported previously.

### The Association of Metabolic Perturbations With Clinical Cardiac Risk Factors

**Figure 4:**
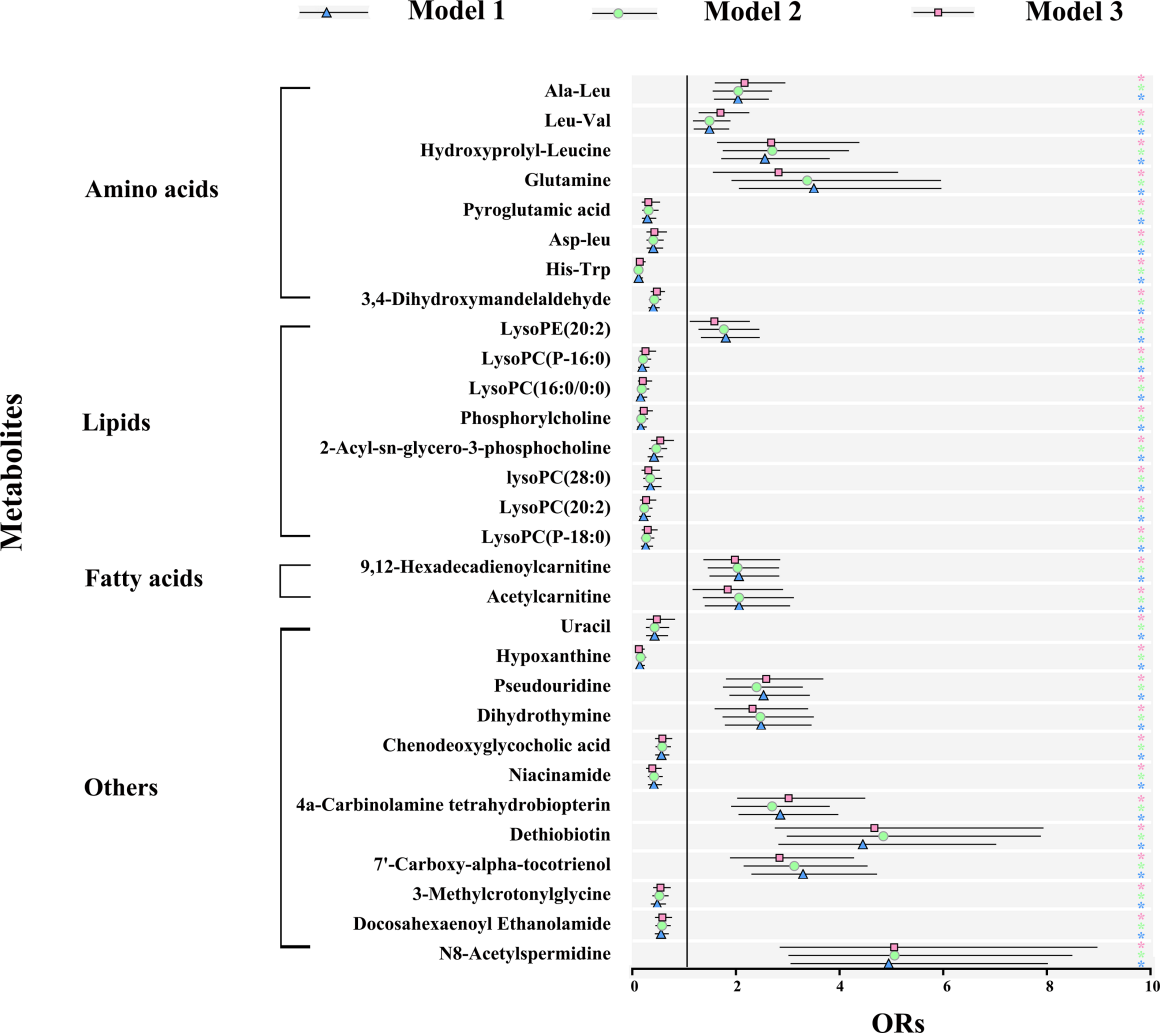
Metabolic signature associations of CAD with clinical risk factors based on results from logistic regression. Model 1 (blue): adjustment for BMI; model 2 (green): adjustment for TC, TG, LDL-C, and HDL-C; model 3 (pink): further adjustment for BMI, TC, TG, HDL-C, LDL-C, SDP, DBP, FGP, HbA1c. Error bars indicate the 95% CI. Significance is indicated (Student-t test). ∗p < 0.05. BMI, body mass index; TC, total cholesterol; TG, triacylglycerol; HDL-C, high-density lipoprotein cholesterol; LDL-C, low-density lipoprotein cholesterol; SBP, systolic blood pressure; DBP, diastolic blood pressure; FPG, fasting plasma glucose; HbA1c, glycosylated hemoglobin.

Logistic regression was performed to evaluate the associations of metabolic perturbations with clinical cardiac risk factors, respectively. As shown in **Figure 4** and **Supplementary Table 3**, blue triangle represented model 1 (adjustment for BMI), green circle represented model 2 (adjustment for combination of TC, TG, LDL-C, and HDL-C), pink square represented model 3 (adjustment for combination of BMI, TC, TG, HDL-C, LDL-C, SDP, DBP, FGP, HbA1c). For the 30 metabolites, all of them remained significant after adjustment for model 1, model 2, and even model 3 (p < 0.05).

For the comparison of MetS *vs.* HCs, all the 26 metabolites were significant after adjustment for model 1 (p < 0.05). On further adjustment for model 2, associations were overall attenuated, but 17 remained significant. And even for model 3, 15 metabolites remained significant (**Supplementary Figure 8** and **Supplementary Table 4**).

In sample size calculation for estimating the metabolic signatures associations of CAD and MetS with clinical risk factors at power of 0.8, alpha level of 0.05 (20), required sample sizes for cohort 1 (metabolites for CAD *vs.* HCs) and cohort 2 (metabolites for MetS *vs.* HCs) are 426 and 21, respectively (**Supplementary Table 5**).

### The Association of the Metabolic Perturbations of CAD and MetS

**Figure 5:**
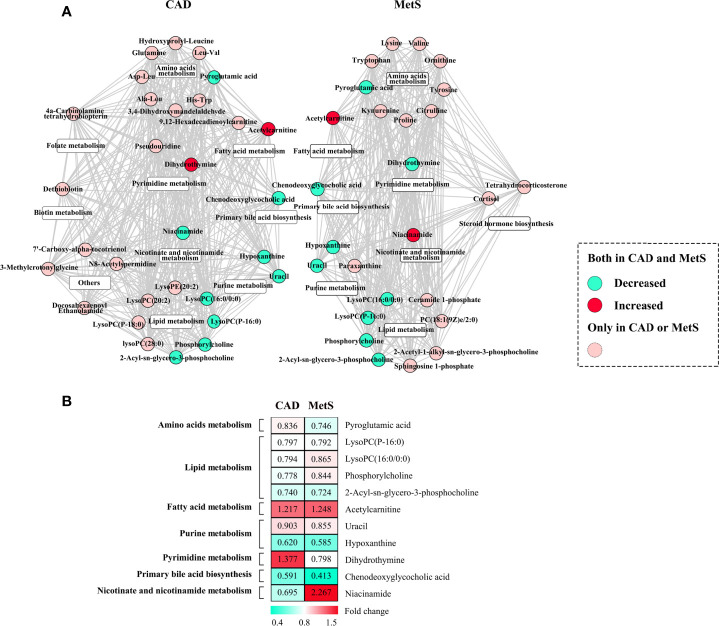
**(A)** Common metabolic signatures between CAD and MetS; **(B)** Fold change analysis of the 12 associated metabolites of CAD and MetS. Green: Downregulated metabolites for CAD or MetS. Red: Upregulated metabolites for CAD or MetS. Pyroglutamic acid, LysoPC (P-16:0), LysoPC (16:0/0:0), phosphorylcholine, 2-Acyl-sn-glycero-3-phosphocholine, uracil, hypoxanthine, chenodeoxyglycocholic acid are downregulated both for CAD and MetS; Acetylcarnitine is upregulated both for CAD and MetS; Dihydrothymine was upregulated for CAD and downregulated for MetS; Ni-acinamide was downregulated for CAD and upregulated for MetS.

Herein, association analysis for the exploration of common metabolic perturbations of CAD and MetS were conducted. The heatmap of correlation coefficients calculated among the significant metabolites of CAD or MetS is shown in **Supplementary Figures 9**, **10**. The metabolites were further subdivided according to the KEGG and HMDB databases (**Figure 5A**). We discovered 11 metabolites both changed for CAD and MetS. Of the 11 metabolites, pyroglutamic acid, LysoPC(P-16:0), LysoPC(16:0/0:0), phosphorylcholine, 2-Acyl-sn-glycero-3-phosphocholine, uracil, hypoxanthine, and chenodeoxyglycocholic acid were downregulated, and acetylcarnitine was upregulated in both CAD and MetS patients. Conversely, dihydrothymine was upregulated in CAD but downregulated in MetS, and niacinamide was downregulated in CAD but upregulated in MetS. As shown in **Figure 5B**, the involved pathways included reduced amino acids metabolism, lipid metabolism, purine metabolism, primary bile acid biosynthesis, and increased fatty acid metabolism. Nevertheless, pyrimidine metabolism and nicotinate and nicotinamide metabolism changed oppositely for CAD and MetS.

### The Risk Predictive Performance of Common Changed Metabolites

**Figure 6:**
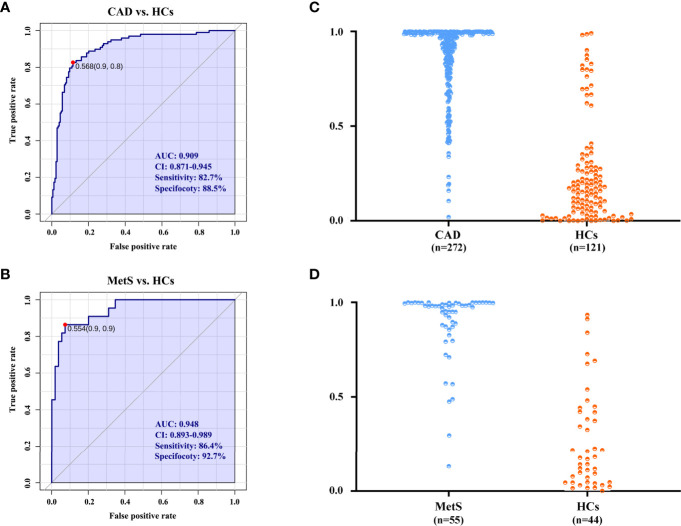
The diagnostic performance is exhibited by the receiver operating characteristic (ROC) curves for CAD and MetS. **(A)** ROC of biomarkers panel for CAD in cohort 1. **(B)** ROC of biomarkers panel for MetS in cohort 2. **(C)** The predictive accuracies of the biomarkers panel in the cohort 1. CAD (n = 272), HCs (n = 121); **(D)** The predictive accuracies of the biomarkers panel in the cohort 2. MetS (n = 55), HCs (n = 44). AUC, area under the curve; CI, confidence interval.

Accurate risk prediction is a prerequisite for effective management of diseases (21). The covariates of nine significant metabolites with same change trend for CAD and MetS were used as biomarkers panel to explore the risk predictive performance, as well as further confirmation of the association of CAD and MetS. For the comparison of CAD *vs.* HCs in the cohort 1, the areas under the AUC curve, sensitivity, and specificity were 0.909, 82.7%, and 88.5% (**Figure 6A**). Predictive value was 86.4% (**Figure 6C**). For the comparison of MetS *vs.* HCs in the cohort 2, the areas under the AUC curve, sensitivity, and specificity were 0.948, 86.4%, and 92.7% (**Figure 6B**), and predictive value was 90.9% (**Figure 6D**). The ROC curve of each metabolite in biomarker panel was shown in **Supplementary Figure 11**. Odds ratios of the biomarker panel and several clinical data were provided in **Supplementary Table 6**.

## Discussion

It is noteworthy that MetS is associated with the risk factors of CAD, which leads to approximately twice occurrence of the CAD event in individuals with MetS (22–25). Nevertheless, the association of CAD and MetS is still only understood partially in humans. Here we report a serum metabolomics profile with UHPLC-Q-Orbitrap HRMS to explore the common metabolic perturbations of CAD and MetS. Impressively, all the 30 identified metabolites for CAD and 15 of 26 metabolites for MetS remained significant after adjustments of combinatorial variables of clinical risk factors.

**Figure 7:**
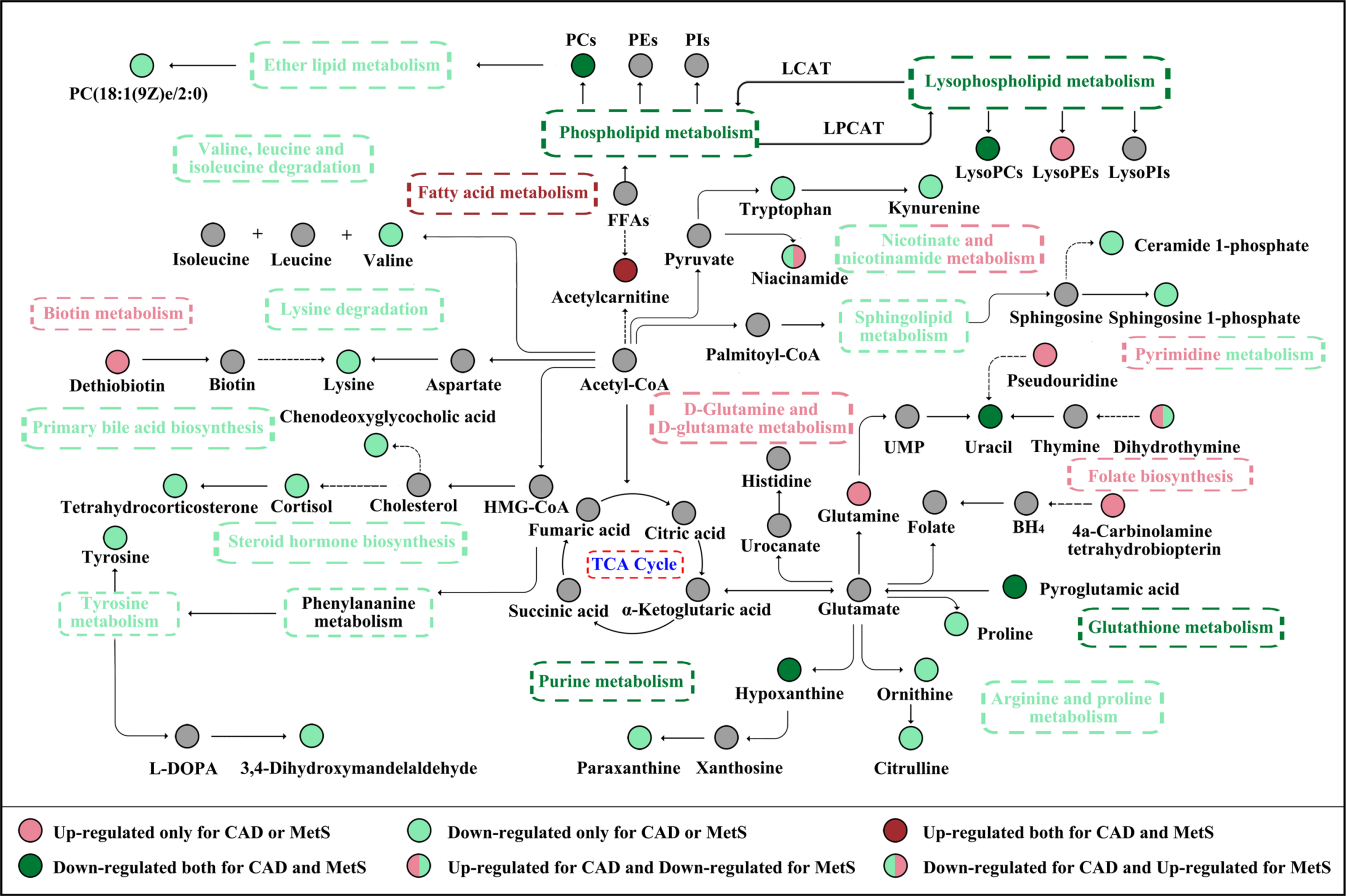
The CAD and MetS-related altered metabolic pathway network of the significantly regulated metabolites. CAD, coronary atherosclerotic disease; MetS, metabolic syndrome.

The association of CAD and MetS has been initially explored. As shown in **Figure 7**, the increased short-chain acylcarnitines levels suggest activated fatty acid metabolism in CAD and MetS (26). Free fatty acid (FFA) could increase the levels of reactive nitrogen species (RNS) and ROS to induce oxidative stress, which lead to β cell dysfunction and insulin resistance by regulating related signaling pathways (27, 28). On the other hand, CAD and MetS patients have downregulated LysoPC(16:0/0:0), LysoPC(P-16:0), phosphorylcholine, and 2-Acyl-sn-glycero-3-phosphocholine. As known, a large number of lysophosphatidylcholines in serum are generated from phosphatidylcholines by the activity of lecithin cholesterol acyltransferase (LCAT). Obviously, low activity of the LCAT has been linked to CAD and MetS (29, 30). The lower pyroglutamic acid related to glutathione metabolism is reduced because CAD and MetS patients have increasing oxidative stress and decreased antioxidant capacity, which lead to lower GSH and higher GSSH (31). The farnesoid X receptor, an endogenous sensor for bile acids, was activated by chenodeoxyglycocholic acid. The inverse correlation has been reported between it and CAD or MetS (32). The altered uracil, hypoxanthine, and dihydrothymine are likely due to the disturbed purine metabolism and pyrimidine metabolism. Uric acid (UA) is the end product of purine metabolism, and either overproduction or underexcretion of UA may cause hyperuricemia (HUA), which is positive correlated to CAD and MetS (33, 34). It has been reported that, by increasing reactive oxygen species (ROS) production in ApoE-knockout mice, hypoxanthine increased the levels of serum cholesterol and the area of atherosclerotic plaque (35). On the other hand, hypoxanthine could aggravate myocardial and renal graft ischemia/reperfusion injury by ROS (36, 37). It is considered that xanthine oxidoreductase (XOR) leads to reduced uracil and hypoxanthine and increased uric acid in our study (38, 39).

As number of water-soluble vitamins, niacinamide is a precursor of NAD and NADP to maintain the cellular metabolisms, including energy metabolism, DNA repair and aging, and oxidative stress. Nevertheless, excessive nicotinamide could exert the toxic effect of MetS by inducing the depletion of free methyl pool, to influence the NAD-dependent enzymatic reaction and trigger oxidative stress (40). Furthermore, niacinamide could be converted to N1-methylnicotinamide (MNAM) by N-methyltransferase (NNMT) (41). It has been reported that MNAM reduces homocysteine secretion by 50%, and elevated homocysteine stimulates atherosclerosis *via* oxidative stress, endothelial dysfunction, and thrombosis and could be regarded as a marker of atherosclerosis (42–44).

Our study has several strengths. First, some significant metabolites, including pseudouridine, dihydrothymine, niacinamide, 4a-Carbinolamine tetrahydrobiopterin for CAD, uracil, hypoxanthine, paraxanthine, dihydrothymine, niacinamide, cortisol, and tetrahydrocorticosterone for MetS, have not been reported elsewhere (11–14, 19). Second, for the first time, the common metabolic perturbations of the pathologic process of CAD and MetS have been performed based on metabolomics profiling, and nine significant metabolites with same change trend have showed excellent risk predictive values (86.4% for CAD and 90.9% for MetS).

Our study also has several limitations. In the current design, the identified metabolite signatures might reflect common perturbation in CAD and MetS attributed to clinical cardiac risk. Nevertheless, prospective cohort-based metabolomics study should be a more proper design to study the specific association between MetS and CAD among those patients after certain follow-up years. Second, the CAD and MetS patients recruited in this study were firstly diagnosed and have no relevant medication history. In addition, the sample size of MetS *vs.* HCs in the cohort 2 is a little small to be sufficiently powered, which needs other replication in an independent cohort with appropriate sample sizes for stratification. Finally, the quantitative study of the altered metabolites is still lacking because of insufficient metabolite standards, and the relative molecular mechanism research to confirm the functions of differential expressed metabolites in MetS and CAD is recommended in future studies.

## Data Availability Statement

The raw data supporting the conclusions of this article will be made available by the authors, without undue reservation.

## Ethics Statement

The studies involving human participants were reviewed and approved by the ethics committee of the First Affiliated Hospital of Zhengzhou University (2019-KY-296). The patients/participants provided their written informed consent to participate in this study. Written informed consent was obtained from the individual(s) for the publication of any potentially identifiable images or data included in this article.

## Author Contributions

Conceptualization, ZJ and LL. Methodology, ZJ, YS, and QD. Investigation and data curation, DZ. Visualization, ZJ, LL, LZ, and SD. Writing—review and editing, ZJ and LL. Project administration, XZ and ZS. All authors contributed to the article and approved the submitted version.

## Funding

This work was supported by the National Natural Science Foundation of China (82003956, 81873188), the Key Scientific Research Project of Colleges and Universities in Henan Province [20A350018], and the joint construction of medical science and technology research projects in Henan Province [LHGJ20190279].

## Conflict of Interest

The authors declare that the research was conducted in the absence of any commercial or financial relationships that could be construed as a potential conflict of interest.

## Publisher’s Note

All claims expressed in this article are solely those of the authors and do not necessarily represent those of their affiliated organizations, or those of the publisher, the editors and the reviewers. Any product that may be evaluated in this article, or claim that may be made by its manufacturer, is not guaranteed or endorsed by the publisher.

## References

[1] Virani SS Alonso A Benjamin EJ Bittencourt MS Callaway CW Carson AP. Heart Disease and Stroke Statistics-2020 Update: A Report From the American Heart Association. Circulation (2020) 141:e139–596. doi: 10.1161/CIR.0000000000000757 31992061

[2] Mortality GBD, Causes of Death C. Global, Regional, and National Age-Sex Specific All-Cause and Cause-Specific Mortality for 240 Causes of Death, 1990-2013: A Systematic Analysis for the Global Burden of Disease Study 2013. Lancet (2015) 385:117–71. doi: 10.1016/S0140-6736(14)61682-2 PMC4340604 25530442

[3] Malik S Wong ND Franklin SS Kamath TV L'Italien GJ Pio JR. Impact of the Metabolic Syndrome on Mortality From Coronary Heart Disease, Cardiovascular Disease, and All Causes in United States Adults. Circulation (2004) 110:1245–50. doi: 10.1161/01.CIR.0000140677.20606.0E 15326067

[4] Wilson PW D'Agostino RB Parise H Sullivan L Meigs JB. Metabolic Syndrome as a Precursor of Cardiovascular Disease and Type 2 Diabetes Mellitus. Circulation (2005) 112:3066–72. doi: 10.1161/CIRCULATIONAHA.105.539528 16275870

[5] Li F Duan J Zhao M Huang S Mu F Su J. A Network Pharmacology Approach to Reveal the Protective Mechanism of Salvia Miltiorrhiza-Dalbergia Odorifera Coupled-Herbs on Coronary Heart Disease. Sci Rep (2019) 9:19343. doi: 10.1038/s41598-019-56050-5 31852981 PMC6920415

[6] Neumann F. 2018 ESC/EACTS Guidelines on Myocardial Revascularization. Rev Esp Cardiol (Engl Ed) (2019) 72:73. doi: 10.1093/eurheartj/ehy394 30580787

[7] Grundy SM Cleeman JI Daniels SR Donato KA Eckel RH Franklin BA. Diagnosis and Management of the Metabolic Syndrome: An American Heart Association/National Heart, Lung, and Blood Institute Scientific Statement. Circulation (2005) 112:2735–52. doi: 10.1161/CIRCULATIONAHA.105.169404 16157765

[8] Saklayen MG. The Global Epidemic of the Metabolic Syndrome. Curr Hypertens Rep (2018) 20:12. doi: 10.1007/s11906-018-0812-z 29480368 PMC5866840

[9] Neitzke U Harder T Plagemann A. Intrauterine Growth Restriction and Developmental Programming of the Metabolic Syndrome: A Critical Appraisal. Microcirculation (2011) 18:304–11. doi: 10.1111/j.1549-8719.2011.00089.x 21418379

[10] Eckel RH Grundy SM Zimmet PZ. The Metabolic Syndrome. Lancet (2005) 365:1415–28. doi: 10.1016/S0140-6736(05)66378-7 15836891

[11] Fan Y Li Y Chen Y Zhao YJ Liu LW Li J. Comprehensive Metabolomic Characterization of Coronary Artery Diseases. J Am Coll Cardiol (2016) 68:1281–93. doi: 10.1016/j.jacc.2016.06.044 27634119

[12] Liu H Chen X Hu X Niu H Tian R Wang H. Alterations in the Gut Microbiome and Metabolism With Coronary Artery Disease Severity. Microbiome (2019) 7:68. doi: 10.1186/s40168-019-0683-9 31027508 PMC6486680

[13] Pujos-Guillot E Brandolini M Petera M Grissa D Joly C Lyan B. Systems Metabolomics for Prediction of Metabolic Syndrome. J Proteome Res (2017) 16:2262–72. doi: 10.1021/acs.jproteome.7b00116 28440083

[14] Rapaport E Bernard R Corday E. Nomenclature and Criteria for Diagnosis of Ischemic Heart Disease. Report of the Joint International Society and Federation of Cardiology/World Health Organization Task Force on Standardization of Clinical Nomenclature. Circulation (1979) 59:607–9. doi: 10.1161/01.CIR.59.3.607 761341

[15] Society. Msrcgo CD. Suggestions on Metabolic Syndrome of Diabetes Branch of Chinese Medical Association. Chin J Diabetes (2004) 12:156–61. doi: CNKI:SUN:ZGTL.0.2004-03-001

[16] Curovic VR Suvitaival T Mattila I Ahonen L Trošt K Theilade S. Circulating Metabolites and Lipids Are Associated to Diabetic Retinopathy in Individuals With Type 1 Diabetes. Diabetes (2020) 69:2217–26. doi: 10.2337/db20-0104 PMC7506826 32737117

[17] Herder C Kowall B Tabak AG Rathmann W. The Potential of Novel Biomarkers to Improve Risk Prediction of Type 2 Diabetes. Diabetologia (2014) 57:16–29. doi: 10.1007/s00125-013-3061-3 24078135

[18] Yengo L Arredouani A Marre M Roussel R Vaxillaire M Falchi M. Impact of Statistical Models on the Prediction of Type 2 Diabetes Using Non-Targeted Metabolomics Profiling. Mol Metab (2016) 5:918–25. doi: 10.1016/j.molmet.2016.08.011 PMC5034686 27689004

[19] Libert DM Nowacki AS Natowicz MR. Metabolomic Analysis of Obesity, Metabolic Syndrome, and Type 2 Diabetes: Amino Acid and Acylcarnitine Levels Change Along a Spectrum of Metabolic Wellness. PeerJ (2018) 6:e5410. doi: 10.7717/peerj.5410 30186675 PMC6120443

[20] Tofte N Suvitaival T Trost K Mattila IM Theilade S Winther SA. Metabolomic Assessment Reveals Alteration in Polyols and Branched Chain Amino Acids Associated With Present and Future Renal Impairment in a Discovery Cohort of 637 Persons With Type 1 Diabetes. Front Endocrinol (Lausanne) (2019) 10:818. doi: 10.3389/fendo.2019.00818 31824430 PMC6883958

[21] Mortensen MB Nordestgaard BG. Statin Use in Primary Prevention of Atherosclerotic Cardiovascular Disease According to 5 Major Guidelines for Sensitivity, Specificity, and Number Needed to Treat. JAMA Cardiol (2019) 4:1131–38. doi: 10.1001/jamacardio.2019.3665 PMC6777225 31577339

[22] Mabasa L Samodien E Sangweni NF Pheiffer C Louw J Johnson R. In Utero One-Carbon Metabolism Interplay and Metabolic Syndrome in Cardiovascular Disease Risk Reduction. Mol Nutr Food Res (2020) 64:e1900377. doi: 10.1002/mnfr.201900377 31408914

[23] O'Neill S O'Driscoll L. Metabolic Syndrome: A Closer Look at the Growing Epidemic and Its Associated Pathologies. Obes Rev (2015) 16:1–12. doi: 10.1111/obr.12229 25407540

[24] Carr MC Brunzell JD. Abdominal Obesity and Dyslipidemia in the Metabolic Syndrome: Importance of Type 2 Diabetes and Familial Combined Hyperlipidemia in Coronary Artery Disease Risk. J Clin Endocrinol Metab (2004) 89:2601–7. doi: 10.1210/jc.2004-0432 15181030

[25] Tofte N Vogelzangs N Mook-Kanamori D Brahimaj A Nano J Ahmadizar F. Plasma Metabolomics Identifies Markers of Impaired Renal Function: A Meta-Analysis of 3089 Persons With Type 2 Diabetes. J Clin Endocrinol Metab (2020) 105:dgaa173. doi: 10.1210/clinem/dgaa173 32271379

[26] Koves TR Ussher JR Noland RC Slentz D Mosedale M Ilkayeva O. Mitochondrial Overload and Incomplete Fatty Acid Oxidation Contribute to Skeletal Muscle Insulin Resistance. Cell Metab (2008) 7:45–56. doi: 10.1016/j.cmet.2007.10.013 18177724

[27] Psichas A Sleeth ML Murphy KG Brooks L Bewick GA Hanyaloglu AC. The Short Chain Fatty Acid Propionate Stimulates GLP-1 and PYY Secretion *via* Free Fatty Acid Receptor 2 in Rodents. Int J Obes (Lond) (2015) 39:424–9. doi: 10.1038/ijo.2014.153 PMC4356745 25109781

[28] Christiansen E Hudson BD Hansen AH Milligan G Ulven T. Development and Characterization of a Potent Free Fatty Acid Receptor 1 (FFA1) Fluorescent Tracer. J Med Chem (2016) 59:4849–58. doi: 10.1021/acs.jmedchem.6b00202 27074625

[29] Ganna A Salihovic S Sundstrom J Broeckling CD Hedman AK Magnusson PK. Large-Scale Metabolomic Profiling Identifies Novel Biomarkers for Incident Coronary Heart Disease. PloS Genet (2014) 10:e1004801. doi: 10.1371/journal.pgen.1004801 25502724 PMC4263376

[30] Stegemann C Pechlaner R Willeit P Langley SR Mangino M Mayr U. Lipidomics Profiling and Risk of Cardiovascular Disease in the Prospective Population-Based Bruneck Study. Circulation (2014) 129:1821–31. doi: 10.1161/CIRCULATIONAHA.113.002500 24622385

[31] Patel RS Ghasemzadeh N Eapen DJ Sher S Arshad S Ko YA. Novel Biomarker of Oxidative Stress Is Associated With Risk of Death in Patients With Coronary Artery Disease. Circulation (2016) 133:361–9. doi: 10.1161/CIRCULATIONAHA.115.019790 PMC4722941 26673559

[32] Lin Z Pan X Wu F Ye D Zhang Y Wang Y. Fibroblast Growth Factor 21 Prevents Atherosclerosis by Suppression of Hepatic Sterol Regulatory Element-Binding Protein-2 and Induction of Adiponectin in Mice. Circulation (2015) 131:1861–71. doi: 10.1161/CIRCULATIONAHA.115.015308 PMC4444420 25794851

[33] Jordheim LP Peters GJ. Recent Updates on Purine and Pyrimidine Metabolism in Physiological and Pathological Settings. Nucleosides Nucleotides Nucleic Acids (2020) 1–8. doi: 10.1080/15257770.2020.1730891 32182157

[34] Baker JF Krishnan E Chen L Schumacher HR. Serum Uric Acid and Cardiovascular Disease: Recent Developments, and Where do They Leave Us? Am J Med (2005) 118:816–26. doi: 10.1016/j.amjmed.2005.03.043 16084170

[35] Fominskiy E Lomivorotov V Nepomniashchikh V Likhvantsev V Ma J De Simone F. Cardiac Protection With Phosphocreatine: A Meta-Analysis. J Cardiothoracic Vasc Anesthesia (2016) 30:S16. doi: 10.1053/j.jvca.2016.03.124 27318357

[36] Abd-Elfattah AS Jessen ME Lekven J Doherty NE 3rd Brunsting LA Wechsler AS. Myocardial Reperfusion Injury. Role of Myocardial Hypoxanthine and Xanthine in Free Radical-Mediated Reperfusion Injury. Circulation (1988) 78:III224–35. 3180402

[37] Domanski L Safranow K Dolegowska B Rózański J Myślak M Ciechanowski K. Hypoxanthine as a Graft Ischemia Marker Stimulates Catalase Activity in the Renal Vein During Reperfusion in Humans. Transplant Proc (2006) 38:35–8. doi: 10.1016/j.transproceed.2005.11.083 16504657

[38] Sjodin B Hellsten Westing Y Apple FS. Biochemical Mechanisms for Oxygen Free Radical Formation During Exercise. Sports Med (1990) 10:236–54. doi: 10.2165/00007256-199010040-00003 2247725

[39] Tani T Okamoto K Fujiwara M Katayama A Tsuruoka S. Metabolomics Analysis Elucidates Unique Influences on Purine / Pyrimidine Metabolism by Xanthine Oxidoreductase Inhibitors in a Rat Model of Renal Ischemia-Reperfusion Injury. Mol Med (2019) 25:40. doi: 10.1186/s10020-019-0109-y 31438839 PMC6704627

[40] Rongvaux A Andris F Van Gool F Leo O. Reconstructing Eukaryotic NAD Metabolism. Bioessays (2003) 25:683–90. doi: 10.1002/bies.10297 12815723

[41] Nejabati HR Mihanfar A Pezeshkian M Fattahi A Latifi Z Safaie N. N1-Methylnicotinamide (MNAM) as a Guardian of Cardiovascular System. J Cell Physiol (2018) 233:6386–94. doi: 10.1002/jcp.26636 29741779

[42] Riederer M Erwa W Zimmermann R Frank S Zechner R. Adipose Tissue as a Source of Nicotinamide N-Methyltransferase and Homocysteine. Atherosclerosis (2009) 204:412–7. doi: 10.1016/j.atherosclerosis.2008.09.015 18996527

[43] Guthikonda S Haynes WG. Homocysteine: Role and Implications in Atherosclerosis. Curr Atheroscler Rep (2006) 8:100–6. doi: 10.1007/s11883-006-0046-4 16510043

[44] Perla-Kajan J Twardowski T Jakubowski H. Mechanisms of Homocysteine Toxicity in Humans. Amino Acids (2007) 32:561–72. doi: 10.1007/s00726-006-0432-9 17285228

