# Supplementary material for: Association of Coronary Artery Disease and Metabolic Syndrome: Usefulness of Serum Metabolomics Approach
Source: Front Endocrinol (Lausanne). 2021 Sep 24;12:692893. doi: 10.3389/fendo.2021.692893 (PMC8498335; doi:10.3389/fendo.2021.692893)
Supplement: Supplementary file 1 [file DataSheet_1.docx]

Supplementary Material

Association of coronary artery disease and metabolic syndrome: usefulness of serum metabolomics approach

**Ziwei Jing ^1,^**^#^**, Liwei Liu ^1,#^, Yingying Shi ^1^, Qiuzheng Du ^1^, Dingding Zhang ^2^, Lihua Zuo ^1^, Shuzhang Du ^1^, Xiaojian Zhang ^1,^*, Zhi Sun ^1,^***

^1^Department of Pharmacy, The First Affiliated Hospital of Zhengzhou University, Zhengzhou, Henan, China

^2^Department of Vasculocardiology, The First Affiliated Hospital of Zhengzhou University, Zhengzhou, Henan, China

*** Correspondence:**Xiaojian Zhang, zhangxiaojian_yxb@163.com; Zhi Sun, [sunzhi2013@163.com](mailto:sunzhi2013@163.com)


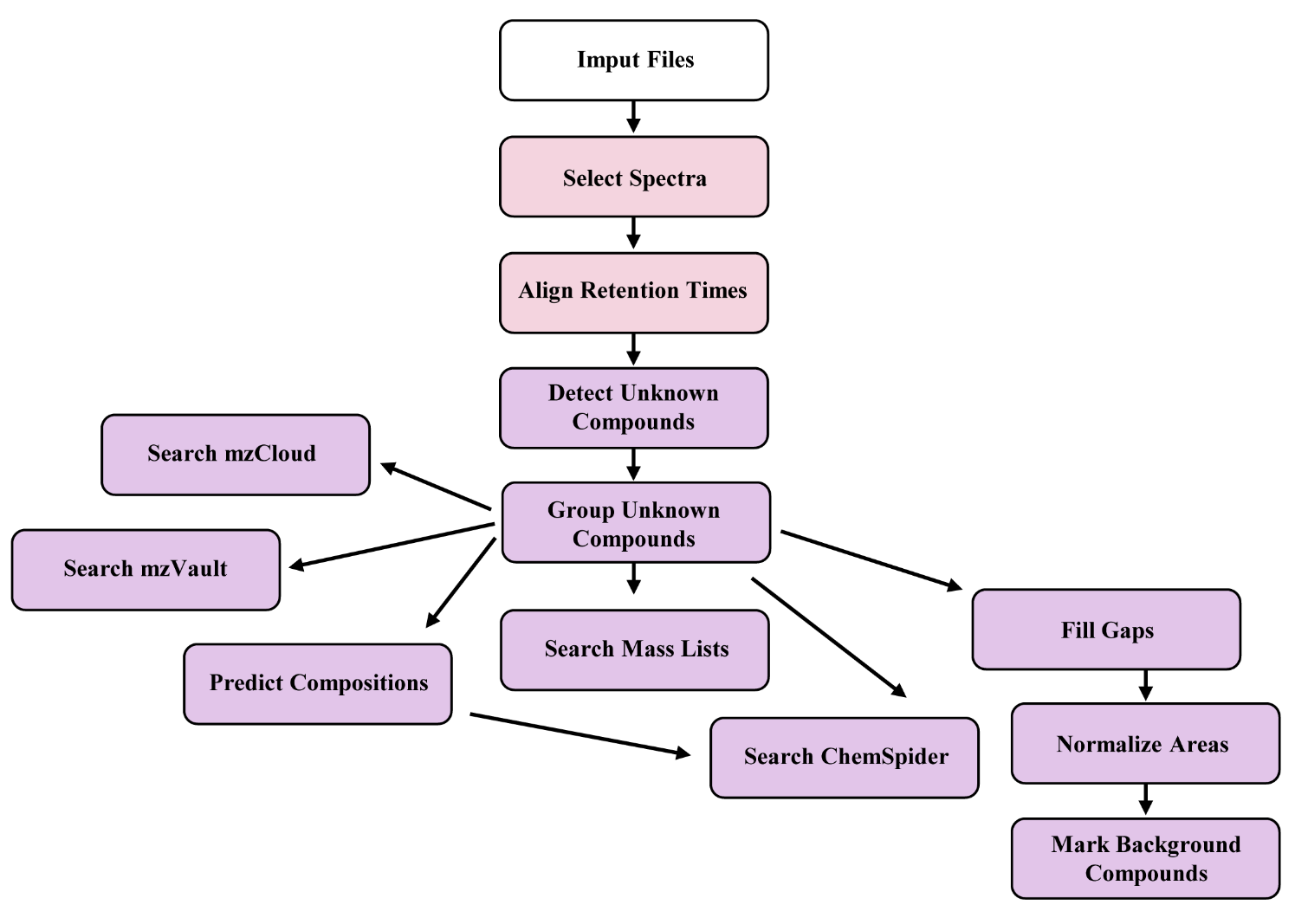


**Supplementary** **Figure. 1.** The flow chart for the non-targeted serum metabolomics procedure. mzVault: a private mass spectral database from Henan Engineering Research Center of Clinical Mass Spectrometry for Precision Medicine; Fill Gaps: Fills the gaps for missing peaks in detected compounds; Normalize Areas” means “Normalizes the area of detected compounds.


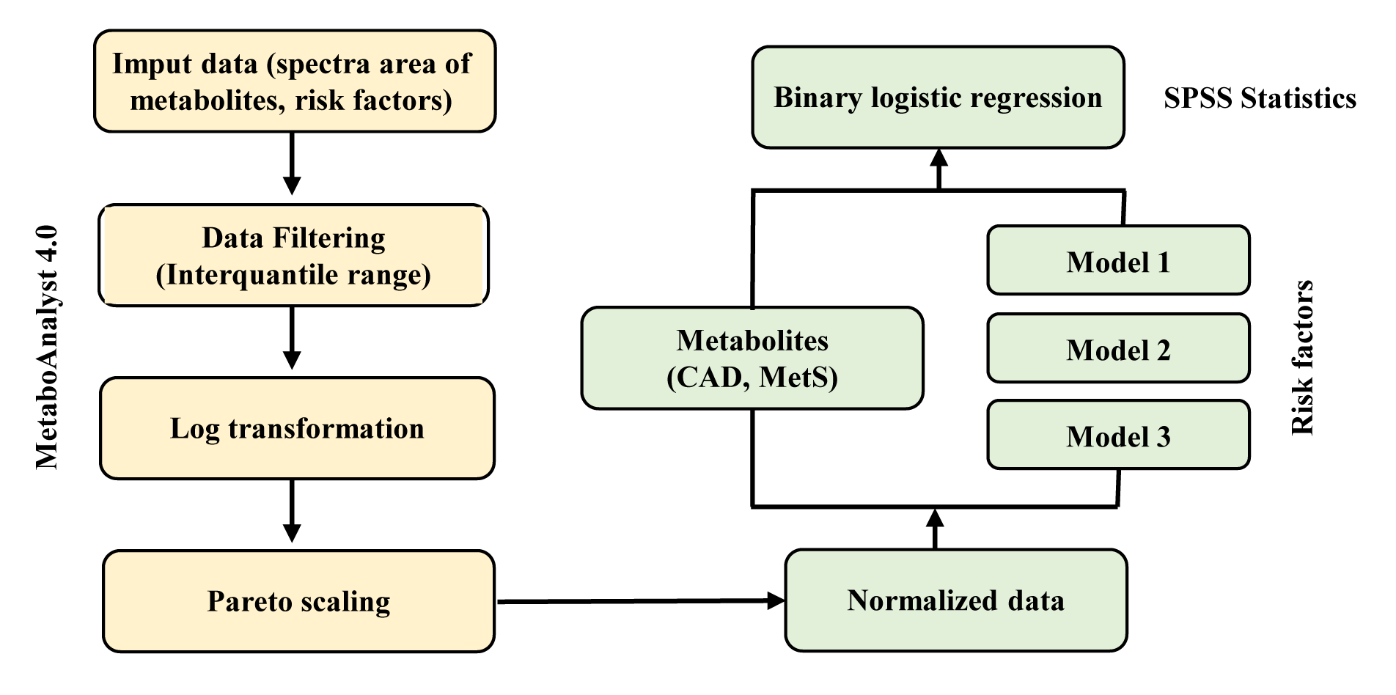


**Supplementary** **Figure. 2.** The logistic regression procedure to investigate the association of significant metabolites for CAD and MetS with clinical cardiac risk factors. Data filtering: to identify and remove variables that are unlikely to be of use when modeling the data; Log transformation: generalized logarithm transformation; Pareto scaling: mean-centered and divided by the square root of the standard deviation of each variable.

**
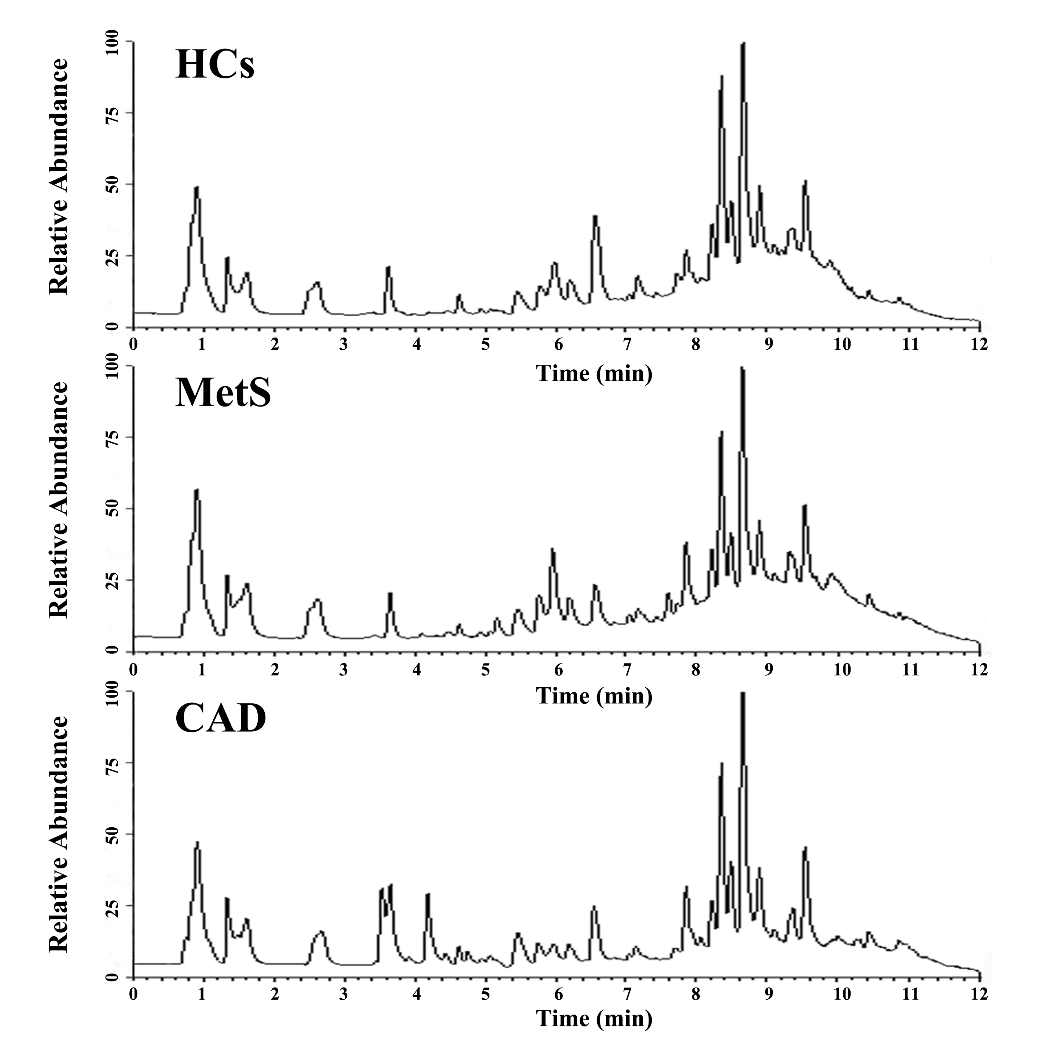
**

**Supplementary** **Figure 3.** Representative total ion chromatograms (TICs) of HCs, MetS and CAD. HCs = Healthy controls (n=165); MetS = Metabolic syndrome (n=55); CAD = Coronary artery disease (n=272).

**
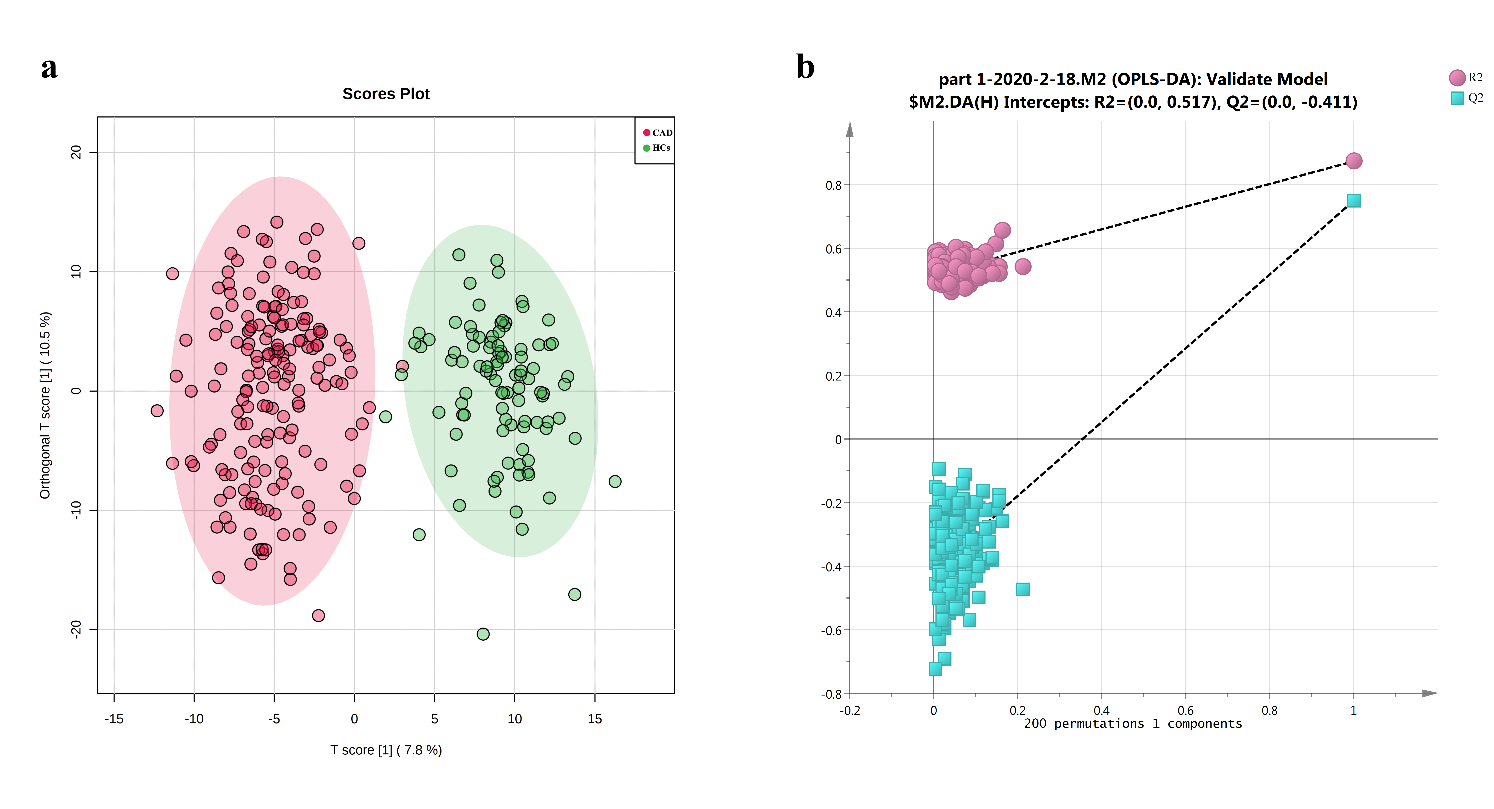
**

**Supplementary** **Figure 4.** Metabolic signatures associated with CAD in cohort 1. (**a**) The orthogonal partial least-squares discriminate analysis for CAD vs. HCs. (**b**) Permutation test was carried to avoid overfitting.


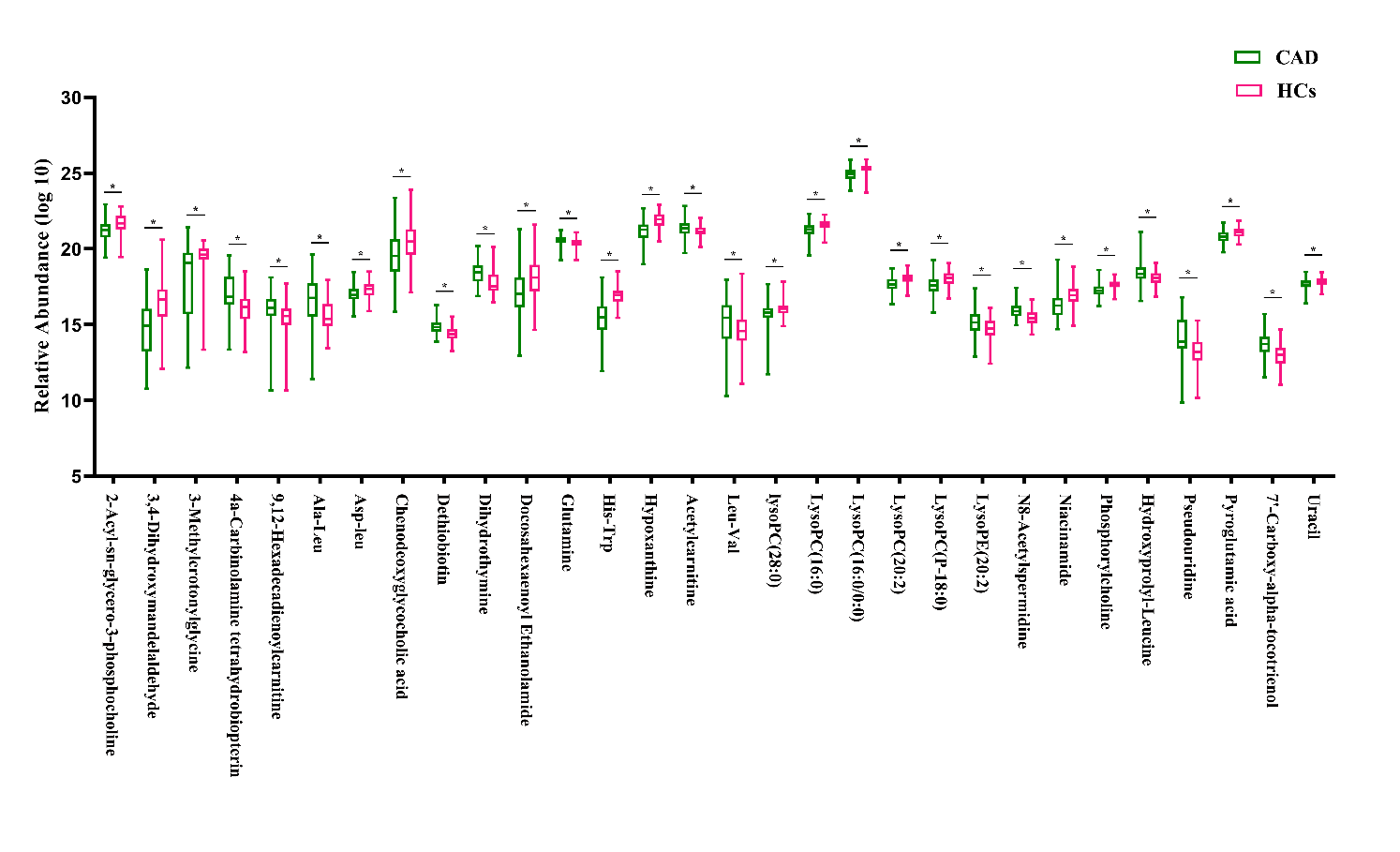


**Supplementary** **Figure. 5.** Identification of the altered metabolites associated with CAD. The box plot shows that the metabolite significantly changed between CAD and HCs. *p < 0.05 by Student-t test, boxes represent the inter-quartile ranges, and lines inside the boxes denote medians.

**
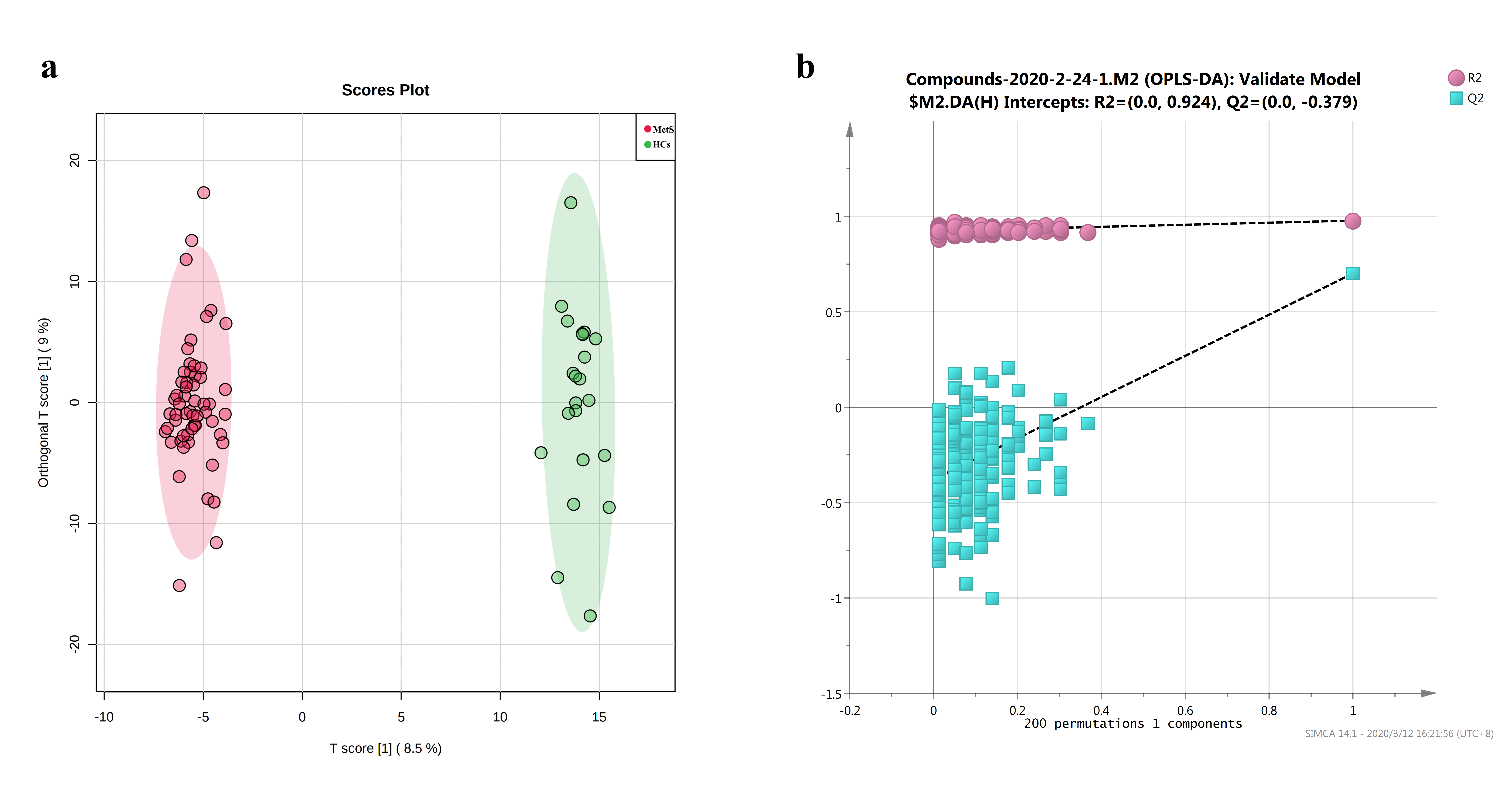
**

**Supplementary** **Figure. 6.** Metabolic signatures associated with MetS in cohort 2. (**a**) The orthogonal partial least-squares discriminate analysis for MetS vs. HCs. (**b**) Permutation test was carried to avoid overfitting.

**
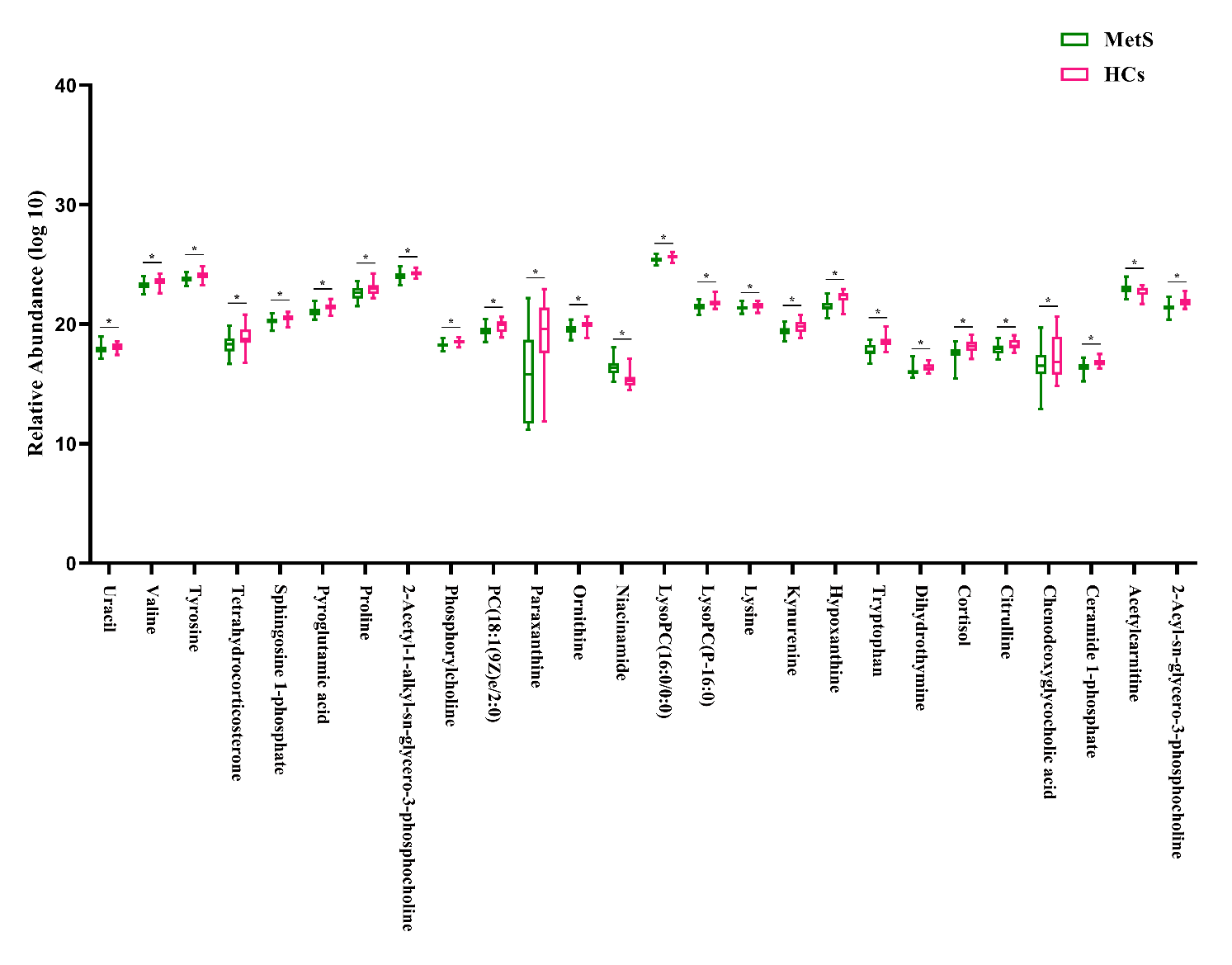
**

**Supplementary** **Figure. 7.** Identification of the altered metabolites associated with MetS. The box plot shows that the metabolite significantly changed between MetS and HCs. *p < 0.05 by Student-t test, boxes represent the inter-quartile ranges, and lines inside the boxes denote medians.


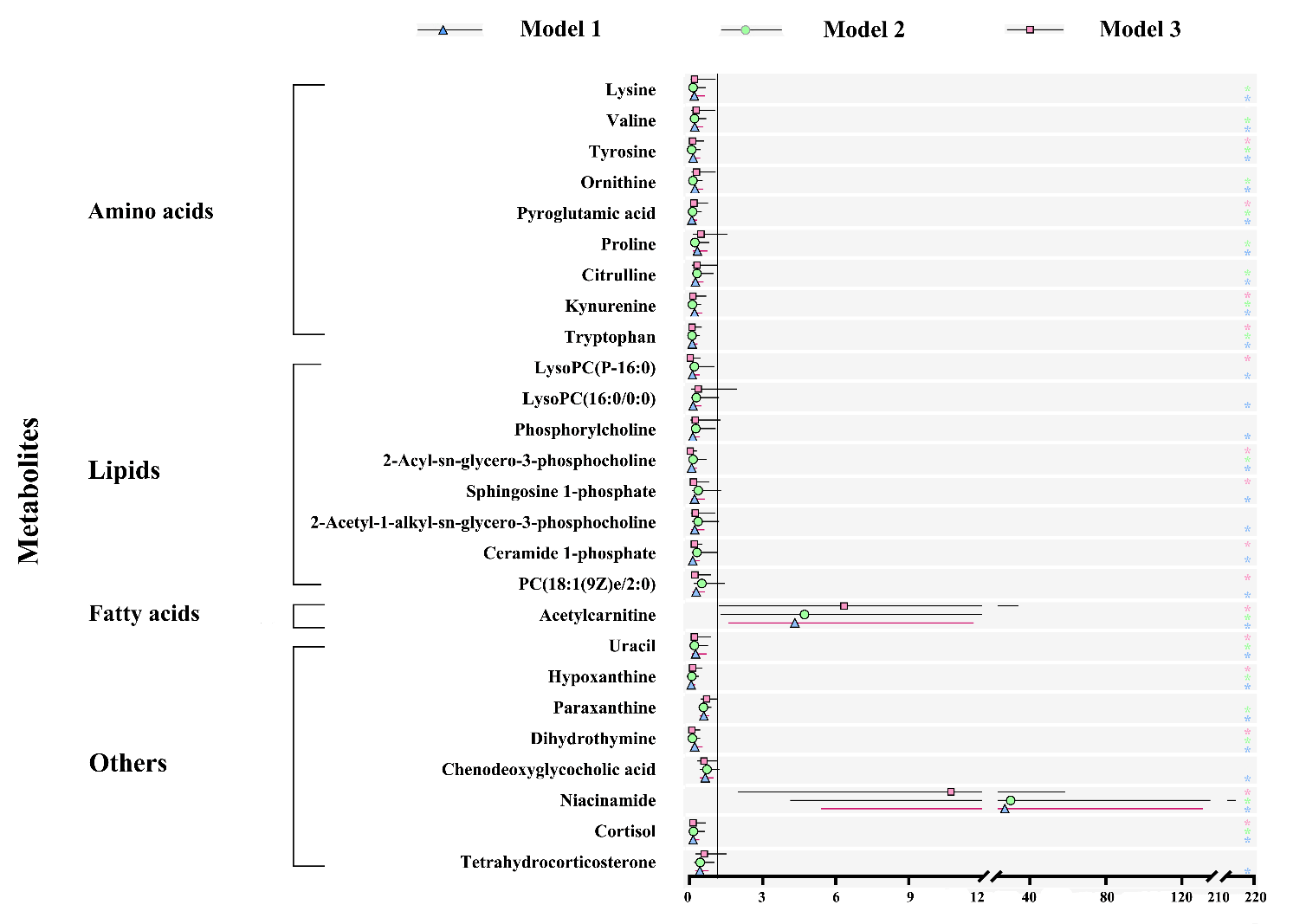


**Supplementary** **Figure. 8.** Metabolic signatures associations of MetS with clinical risk factors based on results from logistic regression. Model 1 (blue): adjustment for BMI; model 2 (green): adjustment for TC, TG, LDL-C and HDL-C; model 3 (pink): further adjustment for BMI, TC, TG, HDL-C, LDL-C, SDP, DBP, FGP, HbA1c. Error bars indicate the 95% CI; Significance is indicated (Student-t test). ∗p < 0.05. BMI = Body mass index; TC = Total cholesterol; TG = Triacylglycerol; HDL-C = High density lipoprotein cholesterol; LDL-C = Low density lipoprotein cholesterol; SBP = Systolic blood pressure; DBP = Diastolic blood pressure; FPG = Fasting plasma glucose; HbA1c = Glycosylated hemoglobin.


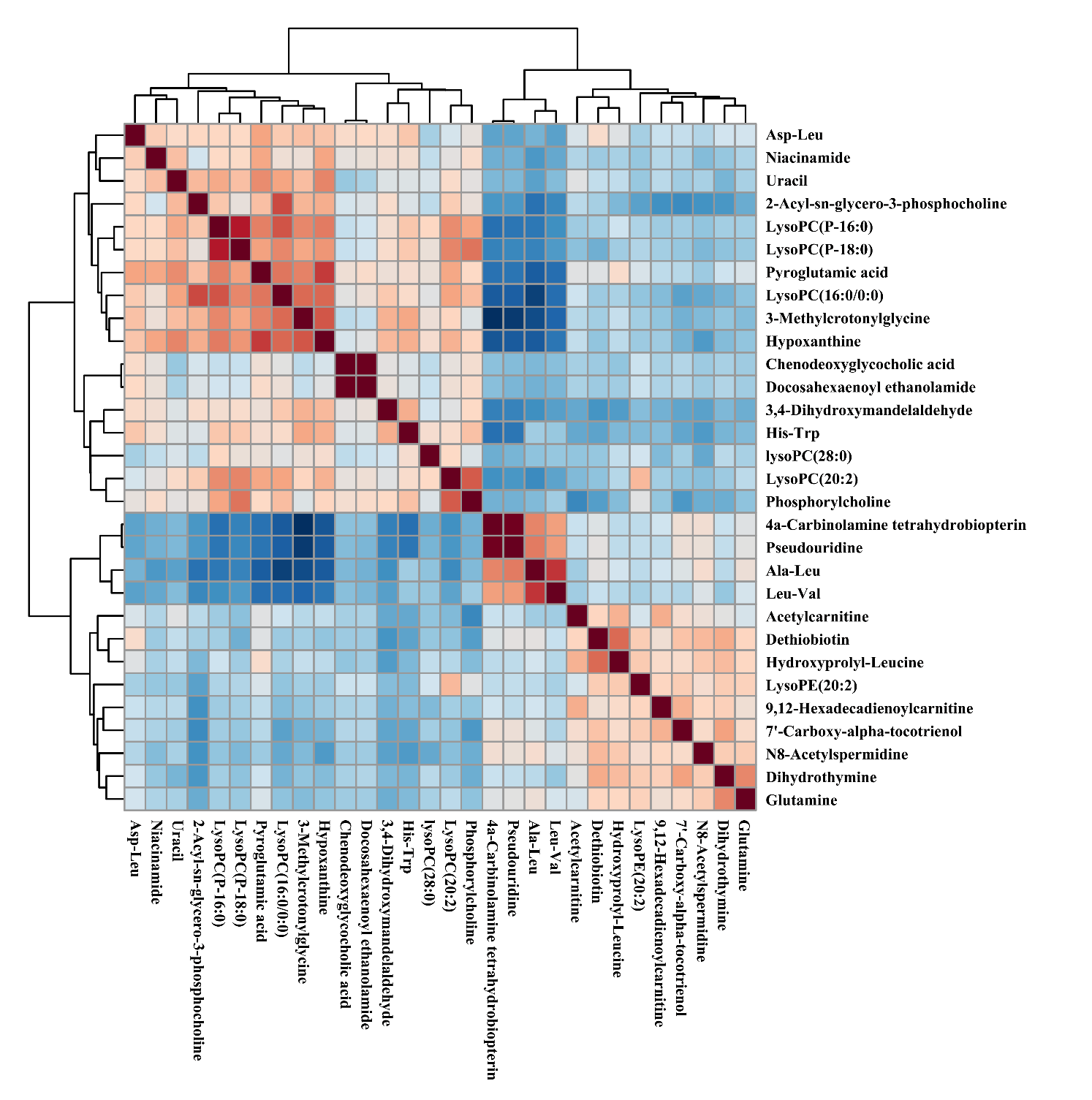


**Supplementary** **Figure. 9.** The heatmap of correlation coefficients calculated among the 30 differential metabolites for CAD in the cohort 1.

**
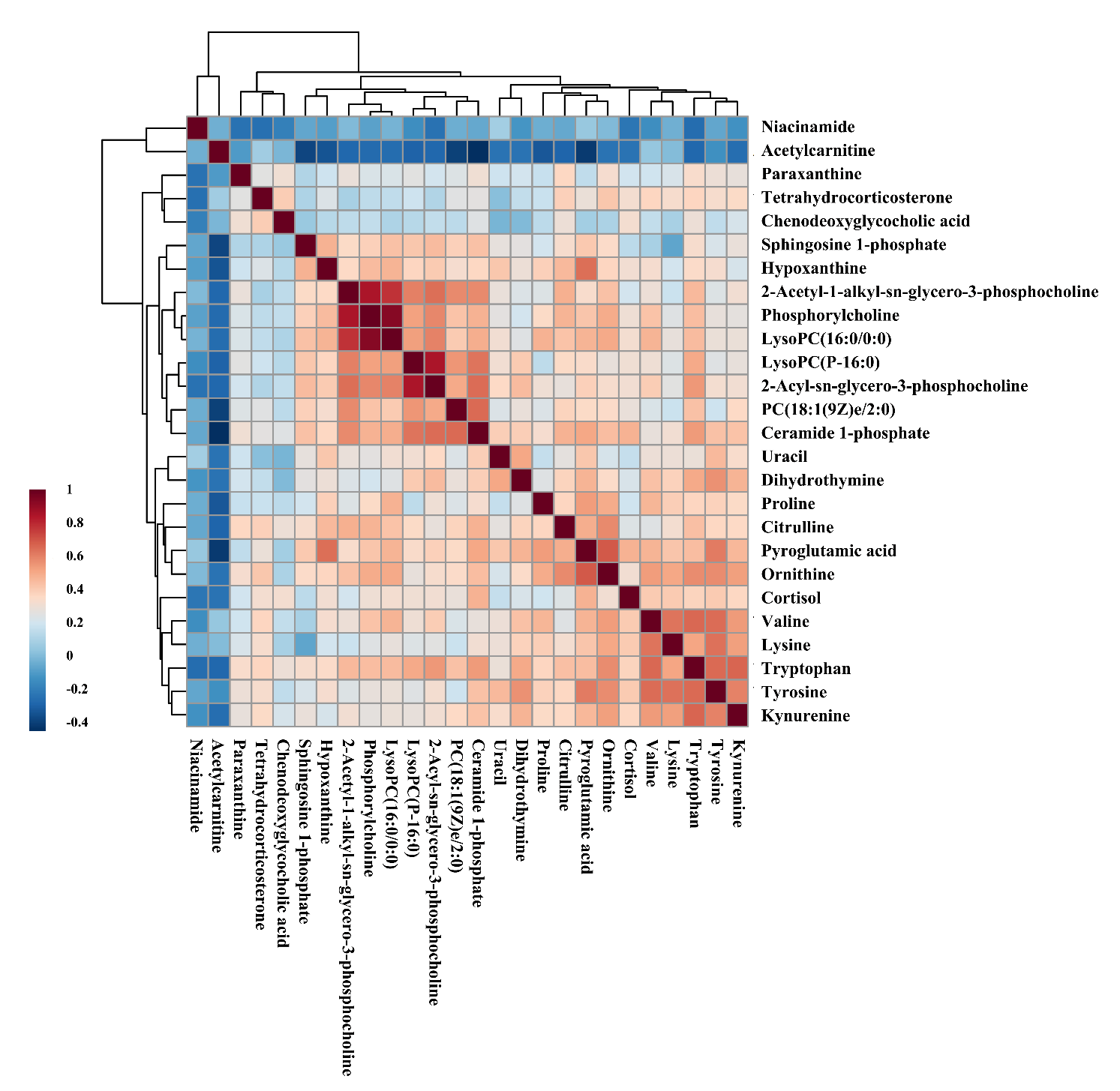
**

**Supplementary** **Figure. 10.** The heatmap of correlation coefficients calculated among the 26 differential metabolites for MetS in the cohort 2.

**
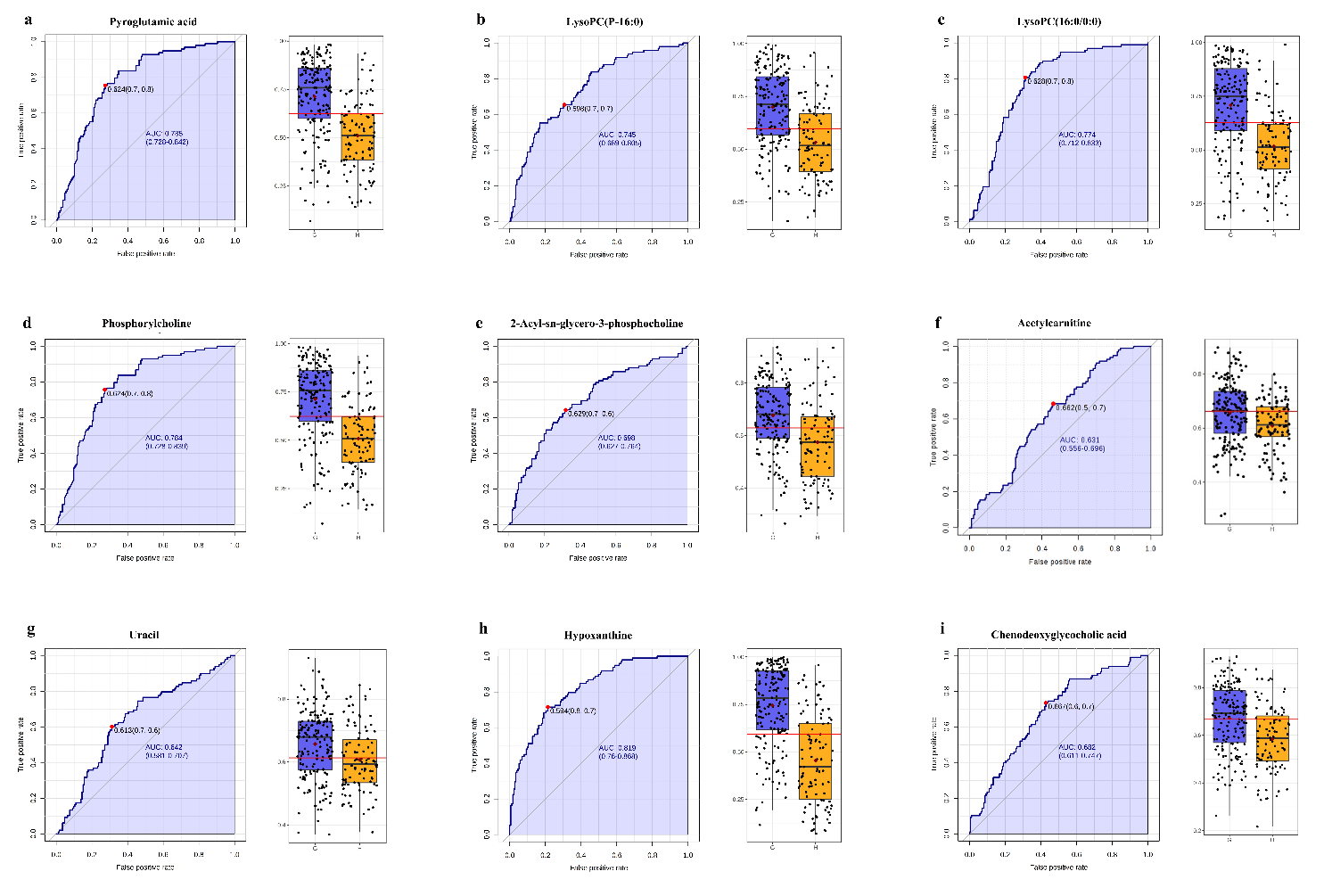
**

**Supplementary** **Figure. 11.** The diagnostic performance of each metabolite in biomarker panel are exhibited by the receiver operating characteristic (ROC) curves for MetS. (**a**) Pyroglutamic acid. (**b**) LysoPC(P-16:0). (**c**) LysoPC(16:0/0:0). (**d**) Phosphorylcholine. (**e**) 2-Acyl-sn-glycero-3-phosphocholine. (**f**) Acetylcarnitine. (**g**) Uracil. (**h**) Hypoxanthine. (**i**) Chenodeoxyglycocholic acid. AUC = area under the curve; CI = confidence interval.

**Supplementary Table. 1.** The details of inclusion and exclusion criteria in this study.

|  | **HCs** | **CAD** | **MetS** |
| --- | --- | --- | --- |
| **INCLUSION CRITARIA** |  |  |  |
| Among 35~85 yrs | √ | √ | √ |
| Simple overweight or obesity and metabolically well | √ |  |  |
| Diagnostic criteria of MetS |  |  | √ |
| Diagnostic criteria of CAD |  | √ |  |
| **EXCLUSION CRITARIA** |  |  |  |
| Infection, fever, trauma, burns, surgery within a week, active tuberculosis or rheumatic immune diseases | √ | √ | √ |
| Severe arrhythmia (atrial fibrillation, atrial flutter, paroxysmal ventricular tachycardia), combined with hemodynamic changes |  | √ |  |
| Chronic heart failure phase C |  | √ |  |
| Patients with CAD | √ |  | √ |
| Valvulopathy or primary cardiomyopathy | √ | √ | √ |
| Serious period of COPD, pulmonary heart disease, respiratory failure | √ | √ | √ |
| Cerebrovascular disease | √ | √ | √ |
| Renal dysfunction (creatinine > 221 μmol/L for men or > 177 μmol/L for women) | √ | √ | √ |
| Liver dysfunction (alanine aminotransferase level >120 U/L) or with cirrhosis | √ | √ | √ |
| Combined with hematological disease or malignant tumor | √ | √ | √ |
| Organ transplant patients | √ | √ | √ |
| Severe mental disorders | √ | √ | √ |
| Women with pregnant or breastfeeding | √ | √ | √ |

HCs = Healthy controls (n=165); CAD = Coronary artery disease (n=272); MetS = Metabolic syndrome (n=55).

**Supplementary Table. 2.** Basic clinical characteristics of participants in this study.

| **Parameters** |  | **Cohort 1** | |  | **Cohort 2** | |  |
| --- | --- | --- | --- | --- | --- | --- | --- |
|  |  | **CAD(n=272)** | **HCs (n=121)** |  | **MetS (n=55)** | **HCs (n=44)** |  |
| Age, yrs |  | 53.4 ± 13.0 | 52.7 ± 10.0 |  | 53.4 ± 9.7 | 52.4 ± 13.7 |  |
| Female, % |  | 49.7 | 49.0 |  | 52.7 | 54.5 |  |
| BMI, kg/m^2^ |  | 25.4 ± 3.8 | 24.5 ± 3.0 |  | 26.2 ± 3.5 | 25.9 ± 2.4 |  |
| **Blood pressure,**  **mm Hg** |  |  |  |  |  |  |  |
| SBP |  | 136.5 ± 10.1^*^ | 128.2 ± 19.0 |  | 133.8 ± 16.1^*^ | 122.3 ± 20.0 |  |
| DBP |  | 89.3 ± 7.9 | 87.2 ± 7.8 |  | 88.9 ± 15.9 | 85.9 ± 20.6 |  |
| **Laboratory data** |  |  |  |  |  |  |  |
| HbA_1c_, % |  | 6.4 ± 0.7^*^ | 5.9 ± 1.0 |  | 6.6 ± 1.7^*^ | 5.9 ± 1.0 |  |
| FPG, mmol/L |  | 6.3 ± 1.2^*^ | 6.0 ± 0.2 |  | 6.5 ± 1.5^*^ | 5.7 ± 3.3 |  |
| ALT, U/L |  | 22.6 ± 4.7 | 22.8 ± 7.7 |  | 22.2 ± 6.4 | 20.9 ± 5.4 |  |
| AST, U/L |  | 19.6 ± 5.0 | 20.2 ± 7.8 |  | 19.3 ± 3.3 | 18.7 ± 7.9 |  |
| TC, mmol/L |  | 4.4 ± 0.6 | 4.2 ± 0.8 |  | 4.3 ± 0.9^*^ | 3.8 ± 1.1 |  |
| TG, mmol/L |  | 1.7 ± 0.7^*^ | 1.5 ± 0.6 |  | 2.0 ± 0.7^*^ | 1.3 ± 0.5 |  |
| HDL-C, mmol/L |  | 1.1 ± 0.5^*^ | 1.3 ± 0.4 |  | 1.1 ± 0.2^*^ | 1.5 ± 0.4 |  |
| LDL-C, mmol/L |  | 2.8 ± 0.4^*^ | 2.6 ± 0.6 |  | 2.8 ± 0.6^*^ | 2.1 ± 0.7 |  |
| eGFR,mL/min/1.73m^2^ |  | 95.0 ± 13.1 | 94.2 ± 13.4 |  | 94.9 ± 9.4 | 96.2 ± 12.5 |  |
| CR, mmol/L |  | 67.5 ± 22.3 | 70.6 ± 12.9 |  | 66.3 ± 15.9 | 72.7 ± 18.3 |  |

Results are expressed as the number (%) or means ±SD, *P<0.05, compared with healthy controls; BMI = Body mass index; SBP/DBP = Systolic/Diastolic; HbA_1c_ = Glycosylated hemoglobin; FPG = Fasting plasma glucose; ALT = Alanine aminotransferase; AST = Glutamic oxaloacetylase; TC = Total cholesterol; TG = Triacylglycerol; HDL-C = High density lipoprotein cholesterol; LDL-C = Low density lipoprotein cholesterol; eGFR = Glomerular filtration rate; CR = Creatinine. Statistical analysis of the differences among the groups was performed with Student-t test.

**Supplementary Table. 3.** Odds ratio of CAD according to metabolites in per standard deviation (SD) among all cases and matched controls.

| **Metabolites** |  | **Fold Change** | **Model** | **Odds ratio (95% CI)** | **p Value** |
| --- | --- | --- | --- | --- | --- |
| **Ala-Leu** | Amino acids | 2.599 | **Model 1** | 2.038(1.577-2.635) | <0.001* |
|  |  |  | **Model 2** | 2.046(1.553-2.697) | <0.001* |
|  |  |  | **Model 3** | 2.167(1.59-2.954) | <0.001* |
| **Leu-Val** |  | 1.565 | **Model 1** | 1.488(1.184-1.87) | 0.001* |
|  |  |  | **Model 2** | 1.490(1.170-1.897) | 0.001* |
|  |  |  | **Model 3** | 1.702(1.281-2.260) | <0.001* |
| **Hydroxyprolyl-Leucine** |  | 1.369 | **Model 1** | 2.559(1.717-3.812) | <0.001* |
|  |  |  | **Model 2** | 2.700(1.745-4.180) | <0.001* |
|  |  |  | **Model 3** | 2.679(1.637-4.383) | <0.001* |
| **Glutamine** |  | 1.139 | **Model 1** | 3.502(2.056-5.965) | <0.001* |
|  |  |  | **Model 2** | 3.377(1.914-5.958) | <0.001* |
|  |  |  | **Model 3** | 2.823(1.554-5.130) | 0.001* |
| **Pyroglutamic acid** |  | 0.836 | **Model 1** | 0.293(0.184-0.465) | <0.001* |
|  |  |  | **Model 2** | 0.313(0.193-0.507) | <0.001* |
|  |  |  | **Model 3** | 0.311(0.181-0.535) | <0.001* |
| **Asp-leu** |  | 0.796 | **Model 1** | 0.406(0.277-0.597) | <0.001* |
|  |  |  | **Model 2** | 0.406(0.272-0.606) | <0.001* |
|  |  |  | **Model 3** | 0.427(0.272-0.670) | <0.001* |
| **His-Trp** |  | 0.447 | **Model 1** | 0.123(0.072-0.208) | <0.001* |
|  |  |  | **Model 2** | 0.119(0.068-0.208) | <0.001* |
|  |  |  | **Model 3** | 0.145(0.081-0.259) | <0.001* |
| **3,4-Dihydroxymandelaldehyde** |  | 0.36 | **Model 1** | 0.406(0.311-0.531) | <0.001* |
|  |  |  | **Model 2** | 0.423(0.321-0.557) | <0.001* |
|  |  |  | **Model 3** | 0.473(0.354-0.633) | <0.001* |
| **LysoPE(20:2)** | Lipids | 1.338 | **Model 1** | 1.805(1.323-2.461) | <0.001* |
|  |  |  | **Model 2** | 1.768(1.275-2.451) | 0.001* |
|  |  |  | **Model 3** | 1.585(1.108-2.269) | 0.012* |
| **LysoPC(P-16:0)** |  | 0.797 | **Model 1** | 0.193(0.113-0.332) | <0.001* |
|  |  |  | **Model 2** | 0.207(0.118-0.365) | <0.001* |
|  |  |  | **Model 3** | 0.254(0.140-0.459) | <0.001* |
| **LysoPC(16:0/0:0)** |  | 0.794 | **Model 1** | 0.161(0.091-0.286) | <0.001* |
|  |  |  | **Model 2** | 0.182(0.100-0.329) | <0.001* |
|  |  |  | **Model 3** | 0.202(0.107-0.382) | <0.001* |
| **Phosphorylcholine** |  | 0.778 | **Model 1** | 0.164(0.095-0.282) | <0.001* |
|  |  |  | **Model 2** | 0.174(0.099-0.305) | <0.001* |
|  |  |  | **Model 3** | 0.222(0.124-0.399) | <0.001* |
| **2-Acyl-sn-glycero-3-phosphocholine** |  | 0.74 | **Model 1** | 0.418(0.295-0.594) | <0.001* |
|  |  |  | **Model 2** | 0.464(0.32-0.672) | <0.001* |
|  |  |  | **Model 3** | 0.539(0.362-0.804) | 0.002* |
| **lysoPC(28:0)** |  | 0.78 | **Model 1** | 0.348(0.216-0.561) | <0.001* |
|  |  |  | **Model 2** | 0.347(0.211-0.570) | <0.001* |
|  |  |  | **Model 3** | 0.309(0.177-0.537) | <0.001* |
| **LysoPC(20:2)** |  | 0.779 | **Model 1** | 0.219(0.134-0.358) | <0.001* |
|  |  |  | **Model 2** | 0.232(0.138-0.390) | <0.001* |
|  |  |  | **Model 3** | 0.264(0.150-0.462) | <0.001* |
| **LysoPC(P-18:0)** |  | 0.728 | **Model 1** | 0.258(0.167-0.401) | <0.001* |
|  |  |  | **Model 2** | 0.268(0.168-0.426) | <0.001* |
|  |  |  | **Model 3** | 0.297(0.180-0.490) | <0.001* |
| **9,12-Hexadecadienoylcarnitine** | Fatty acids | 1.498 | **Model 1** | 2.055(1.488-2.837) | <0.001* |
|  |  |  | **Model 2** | 2.031(1.456-2.833) | <0.001* |
|  |  |  | **Model 3** | 1.979(1.372-2.856) | <0.001* |
| **Acetylcarnitine** |  | 1.217 | **Model 1** | 2.062(1.396-3.046) | <0.001* |
|  |  |  | **Model 2** | 2.060(1.361-3.118) | 0.001* |
|  |  |  | **Model 3** | 1.841(1.164-2.911) | 0.009* |
| **Uracil** | Others | 0.903 | **Model 1** | 0.429(0.266-0.692) | 0.001* |
|  |  |  | **Model 2** | 0.431(0.261-0.713) | 0.001* |
|  |  |  | **Model 3** | 0.473(0.271-0.827) | 0.001* |
| **Hypoxanthine** |  | 0.62 | **Model 1** | 0.144(0.086-0.242) | <0.001* |
|  |  |  | **Model 2** | 0.159(0.094-0.270) | <0.001* |
|  |  |  | **Model 3** | 0.128(0.068-0.240) | <0.001* |
| **Pseudouridine** |  | 2.576 | **Model 1** | 2.534(1.873-3.428) | <0.001* |
|  |  |  | **Model 2** | 2.399(1.749-3.290) | <0.001* |
|  |  |  | **Model 3** | 2.583(1.809-3.687) | <0.001* |
| **Dihydrothymine** |  | 1.377 | **Model 1** | 2.484(1.785-3.458) | <0.001* |
|  |  |  | **Model 2** | 2.471(1.743-3.502) | <0.001* |
|  |  |  | **Model 3** | 2.321(1.587-3.394) | <0.001* |
| **Chenodeoxyglycocholic acid** |  | 0.591 | **Model 1** | 0.563(0.442-0.716) | <0.001* |
|  |  |  | **Model 2** | 0.577(0.447-0.746) | <0.001* |
|  |  |  | **Model 3** | 0.582(0.439-0.772) | <0.001 |
| **Niacinamide** |  | 0.695 | **Model 1** | 0.416(0.302-0.573) | <0.001* |
|  |  |  | **Model 2** | 0.418(0.298-0.587) | <0.001* |
|  |  |  | **Model 3** | 0.388(0.265-0.566) | <0.001* |
| **4a-Carbinolamine tetrahydrobiopterin** |  | 2.597 | **Model 1** | 2.855(2.051-3.976) | <0.001* |
|  |  |  | **Model 2** | 2.696(1.907-3.812) | <0.001* |
|  |  |  | **Model 3** | 3.017(2.025-4.495) | <0.001* |
| **Dethiobiotin** |  | 1.363 | **Model 1** | 4.45(2.819-7.0240) | <0.001* |
|  |  |  | **Model 2** | 4.846(2.979-7.885) | <0.001* |
|  |  |  | **Model 3** | 4.671(2.750-7.936) | <0.001* |
| **7'-Carboxy-alpha-tocotrienol** |  | 1.681 | **Model 1** | 3.294(2.298-4.724) | <0.001* |
|  |  |  | **Model 2** | 3.125(2.150-4.542) | <0.001* |
|  |  |  | **Model 3** | 2.841(1.886-4.280) | <0.001* |
| **3-Methylcrotonylglycine** |  | 0.687 | **Model 1** | 0.482(0.357-0.651) | <0.001* |
|  |  |  | **Model 2** | 0.522(0.388-0.702) | <0.001* |
|  |  |  | **Model 3** | 0.545(0.401-0.741) | <0.001* |
| **Docosahexaenoyl Ethanolamide** |  | 0.568 | **Model 1** | 0.554(0.435-0.705) | <0.001* |
|  |  |  | **Model 2** | 0.575(0.446-0.741) | <0.001* |
|  |  |  | **Model 3** | 0.579(0.437-0.766) | <0.001* |
| **N8-Acetylspermidine** |  | 1.438 | **Model 1** | 4.948(3.052-8.021) | <0.001* |
|  |  |  | **Model 2** | 5.059(3.014-8.494) | <0.001* |
|  |  |  | **Model 3** | 5.053(2.844-8.978) | <0.001* |

Model 1: BMI; Model 2: TC, TG, LDL-C and HDL-C; Model 3: BMI, TC, TG, HDL-C, LDL-C, SDP, DBP, FGP, HbA1c. Error bars indicate the 95% CI; Significance is indicated ((Student-t test). ∗p < 0.05.

**Supplementary Table. 4.** Odds ratio of MetS according to metabolites in per standard deviation (SD) among all cases and matched controls.

| **Metabolites** |  | **Fold Change** | **Model** | **Odds ratio**  **(95% CI)** | **p Value** |
| --- | --- | --- | --- | --- | --- |
| **Lysine** | Amino acids | 0.884 | **Model 1** | 0.194(0.059-0.633) | 0.007* |
|  |  |  | **Model 2** | 0.143(0.031-0.661) | 0.013* |
|  |  |  | **Model 3** | 0.203(0.039-1.073) | 0.060 |
| **Valine** |  | 0.797 | **Model 1** | 0.210(0.078-0.561) | 0.002* |
|  |  |  | **Model 2** | 0.200(0.058-0.687) | 0.011* |
|  |  |  | **Model 3** | 0.269(0.069-1.049) | 0.059 |
| **Tyrosine** |  | 0.796 | **Model 1** | 0.140(0.045-0.436) | 0.001* |
|  |  |  | **Model 2** | 0.087(0.017-0.449) | 0.004* |
|  |  |  | **Model 3** | 0.122(0.025-0.595) | 0.009* |
| **Ornithine** |  | 0.77 | **Model 1** | 0.214(0.081-0.565) | 0.002* |
|  |  |  | **Model 2** | 0.135(0.034-0.541) | 0.005* |
|  |  |  | **Model 3** | 0.284(0.075-1.071) | 0.063 |
| **Pyroglutamic acid** |  | 0.746 | **Model 1** | 0.093(0.027-0.321) | <0.001* |
|  |  |  | **Model 2** | 0.118(0.028-0.502) | 0.004* |
|  |  |  | **Model 3** | 0.180(0.043-0.759) | 0.019* |
| **Proline** |  | 0.763 | **Model 1** | 0.321(0.141-0.729) | 0.007* |
|  |  |  | **Model 2** | 0.219(0.059-0.812) | 0.023* |
|  |  |  | **Model 3** | 0.464(0.139-1.550) | 0.212 |
| **Citrulline** |  | 0.764 | **Model 1** | 0.237(0.098-0.572) | 0.001* |
|  |  |  | **Model 2** | 0.305(0.095-0.977) | 0.045* |
|  |  |  | **Model 3** | 0.304(0.082-1.132) | 0.076 |
| **Kynurenine** |  | 0.722 | **Model 1** | 0.201(0.078-0.520) | 0.001* |
|  |  |  | **Model 2** | 0.115(0.028-0.471) | 0.003* |
|  |  |  | **Model 3** | 0.137(0.028-0.686) | 0.016* |
| **Tryptophan** |  | 0.601 | **Model 1** | 0.105(0.034-0.323) | <0.001* |
|  |  |  | **Model 2** | 0.096(0.021-0.430) | 0.002* |
|  |  |  | **Model 3** | 0.101(0.020-0.504) | 0.005* |
| **LysoPC(P-16:0)** | Lipids | 0.792 | **Model 1** | 0.115(0.031-0.423) | 0.001* |
|  |  |  | **Model 2** | 0.195(0.037-1.012) | 0.052 |
|  |  |  | **Model 3** | 0.025(0.001-0.442) | 0.012* |
| **LysoPC(16:0/0:0)** |  | 0.865 | **Model 1** | 0.142(0.041-0.485) | 0.002* |
|  |  |  | **Model 2** | 0.275(0.062-1.214) | 0.088 |
|  |  |  | **Model 3** | 0.357(0.065-1.945) | 0.234 |
| **Phosphorylcholine** |  | 0.844 | **Model 1** | 0.127(0.038-0.425) | 0.001* |
|  |  |  | **Model 2** | 0.254(0.060-1.071) | 0.062 |
|  |  |  | **Model 3** | 0.233(0.043-1.273) | 0.093 |
| **2-Acyl-sn-glycero-3-phosphocholine** |  | 0.724 | **Model 1** | 0.085(0.024-0.296) | <0.001* |
|  |  |  | **Model 2** | 0.146(0.031-0.695) | 0.016* |
|  |  |  | **Model 3** | 0.022(0.002-0.298) | 0.004* |
| **Sphingosine 1-phosphate** |  | 0.848 | **Model 1** | 0.202(0.066-0.617) | 0.005* |
|  |  |  | **Model 2** | 0.358(0.099-1.294) | 0.117 |
|  |  |  | **Model 3** | 0.160(0.032-0.802) | 0.026* |
| **2-Acetyl-1-alkyl-sn-glycero-3-phosphocholine** |  | 0.845 | **Model 1** | 0.211(0.073-0.609) | 0.004* |
|  |  |  | **Model 2** | 0.348(0.100-1.213) | 0.098 |
|  |  |  | **Model 3** | 0.228(0.049-1.054) | 0.058 |
| **Ceramide 1-phosphate** |  | 0.771 | **Model 1** | 0.131(0.040-0.429) | 0.001* |
|  |  |  | **Model 2** | 0.305(0.081-1.141) | 0.078 |
|  |  |  | **Model 3** | 0.196(0.074-0.516) | 0.001* |
| **PC(18:1(9Z)e/2:0)** |  | 0.759 | **Model 1** | 0.266(0.112-0.632) | 0.003* |
|  |  |  | **Model 2** | 0.499(0.173-1.442) | 0.199 |
|  |  |  | **Model 3** | 0.218(0.054-0.881) | 0.033* |
| **Acetyl-L-carnitine** | Fatty acids | 1.248 | **Model 1** | 4.320(1.600-11.665) | 0.004* |
|  |  |  | **Model 2** | 4.716(1.278-17.405) | 0.020* |
|  |  |  | **Model 3** | 6.339(1.186-33.894) | 0.031* |
| **Uracil** | Others | 0.855 | **Model 1** | 0.244(0.084-0.708) | 0.009* |
|  |  |  | **Model 2** | 0.192(0.049-0.758) | 0.018* |
|  |  |  | **Model 3** | 0.196(0.043-0.891) | 0.035* |
| **Hypoxanthine** |  | 0.585 | **Model 1** | 0.065(0.019-0.220) | <0.001* |
|  |  |  | **Model 2** | 0.089(0.021-0.381) | 0.001* |
|  |  |  | **Model 3** | 0.118(0.027-0.523) | 0.005* |
| **Paraxanthine** |  | 0.249 | **Model 1** | 0.579(0.416-0.807) | 0.001* |
|  |  |  | **Model 2** | 0.567(0.360-0.893) | 0.014* |
|  |  |  | **Model 3** | 0.701(0.441-1.112) | 0.131 |
| **Dihydrothymine** |  | 0.798 | **Model 1** | 0.207(0.080-0.537) | 0.001* |
|  |  |  | **Model 2** | 0.112(0.029-0.433) | 0.002* |
|  |  |  | **Model 3** | 0.088(0.018-0.439) | 0.003* |
| **Chenodeoxyglycocholic acid** |  | 0.413 | **Model 1** | 0.643(0.417-0.991) | 0.045* |
|  |  |  | **Model 2** | 0.710(0.410-1.230) | 0.222 |
|  |  |  | **Model 3** | 0.591(0.312-1.117) | 0.105 |
| **Niacinamide** |  | 2.267 | **Model 1** | 26.603(5.404-130.974) | <0.001* |
|  |  |  | **Model 2** | 29.675(4.131-213.15) | 0.001* |
|  |  |  | **Model 3** | 10.734(1.974-58.379) | 0.006* |
| **Cortisol** |  | 0.646 | **Model 1** | 0.139(0.049-0.400) | <0.001* |
|  |  |  | **Model 2** | 0.156(0.039-0.615) | 0.008* |
|  |  |  | **Model 3** | 0.140(0.030-0.656) | 0.013* |
| **Tetrahydrocorticosterone** |  | 0.599 | **Model 1** | 0.421(0.225-0.790) | 0.007* |
|  |  |  | **Model 2** | 0.434(0.185-1.017) | 0.055 |
|  |  |  | **Model 3** | 0.601(0.238-1.514) | 0.280 |

Model 1: BMI; Model 2: TC, TG, LDL-C and HDL-C; Model 3: BMI, TC, TG, HDL-C, LDL-C, SDP, DBP, FGP, HbA1c. Error bars indicate the 95% CI; Significance is indicated (Student-t test). ∗p < 0.05.

**Supplementary Table. 5.** Calculation of the samples number needed for replicating the metabolic

signatures associations of CAD and MetS with clinical risk factors.

| Group | Power, 1−β | Type I error rate, α | Sample Size |
| --- | --- | --- | --- |
| Cohort 1 | 0.8 | 5% | 246 |
| Cohort 2 | 0.8 | 5% | 21 |

Cohort 1: Metabolites for CAD vs.HCs compared with model 1, model 2 and model 3; Cohort 2: Metabolites

for MetS vs.HCs compared with model 1, model 2 and model 3.

**Supplementary Table. 6.** Logistic regression analysis reveals serum metabolomics signature for the different diagnosis in Cohort 1 and Cohort 2.

|  | **Odds ratio (95% CI)** | **S.E.** | **p value** |
| --- | --- | --- | --- |
| **Comparison I: CAD vs. HCs for Cohort 1** | | | |
| Biomarker Panel | 1928.719 (254.687-14606.01) | 1.033 | <0.01 |
| Age | 0.779 (0.273-2.223) | 0.535 | 0.641 |
| BMI | 0.981 (0.308-3.131) | 0.592 | 0.974 |
| SBP | 10.789 (2.035-57.206) | 0.851 | 0.005 |
| DBP | 1.877 (0.384-9.187) | 0.810 | 0.437 |
| HbA_1c_ | 9.463 (2.502-35.794) | 0.679 | 0.001 |
| FPG | 0.593 (0.153-2.3) | 0.691 | 0.450 |
| ALT | 0.8 (0.348-1.839) | 0.425 | 0.600 |
| AST | 0.885 (0.484-1.618) | 0.308 | 0.692 |
| TC | 1.503 (0.523-4.323) | 0.539 | 0.450 |
| TG | 1.055 (0.557-1.998) | 0.326 | 0.870 |
| HDL-C | 0.774 (0.347-1.724) | 0.409 | 0.530 |
| LDL-C | 2.403 (0.755-7.654) | 0.591 | 0.138 |
| eGFR | 0.884 (0.26-2.997) | 0.623 | 0.843 |
| CR | 0.386 (0.178-0.839) | 0.396 | 0.016 |
| **Comparison Ⅱ: MetS vs. HCs for Cohort 2** | | | |
| Biomarker Panel | 519.089 (106.04-2541.052) | 0.810 | <0.01 |
| Age | 0.606 (0.284-1.292) | 0.387 | 0.195 |
| BMI | 1.85 (0.734-4.663) | 0.472 | 0.192 |
| SBP | 13.787 (3.71-51.243) | 0.670 | <0.01 |
| DBP | 2.655 (0.81-8.697) | 0.605 | 0.107 |
| HbA1c | 4.992 (1.857-13.423) | 0.505 | 0.001 |
| FPG | 1.797 (0.623-5.187) | 0.541 | 0.279 |
| ALT | 0.859 (0.413-1.786) | 0.373 | 0.684 |
| AST | 0.854 (0.508-1.434) | 0.265 | 0.550 |
| TC | 2.133 (0.924-4.926) | 0.427 | 0.076 |
| TG | 1.552 (0.921-2.617) | 0.267 | 0.099 |
| HDL-C | 0.9 (0.505-1.604) | 0.295 | 0.721 |
| LDL-C | 2.25 (0.933-5.43) | 0.449 | 0.071 |
| eGFR | 1.189 (0.468-3.025) | 0.476 | 0.716 |
| CR | 0.451 (0.234-0.871) | 0.336 | 0.018 |

BMI = Body mass index; SBP = Systolic blood pressure; DBP = Diastolic blood pressure; HbA_1c_ = Glycosylated hemoglobin; FPG = Fasting plasma glucose; ALT = Alanine aminotransferase; AST = Glutamic oxaloacetylase; TC = Total cholesterol; TG = Triacylglycerol; HDL-C = High density lipoprotein cholesterol; LDL-C = Low density lipoprotein cholesterol; eGFR = Glomerular filtration rate; CR = Creatinine.
